# Alleviative effects of fluoxetine on depressive**-**like behaviors by epigenetic regulation of BDNF gene transcription in mouse model of post-stroke depression

**DOI:** 10.1038/s41598-017-13929-5

**Published:** 2017-11-02

**Authors:** Hui-Juan Jin, Lei Pei, Ya-Nan Li, Hui Zheng, Shuai Yang, Yan Wan, Ling Mao, Yuan-Peng Xia, Quan-Wei He, Man Li, Zhen-Yu Yue, Bo Hu

**Affiliations:** 1 Department of Neurology, Union Hospital, Tongji Medical College, Huazhong University of Science and Technology, Wuhan, 430022 China; 2 Department of Neurobiology, School of Basic Medicine, Tongji Medical College, Huahzong University of Science and Technology, Wuhan, 430030 China; 3 The Institute for Brain Research (IBR), Collaborative Innovation Center for Brain Science, Huazhong University of Science and Technology, Wuhan, 430030 China; 4 Department of Radiology, Union Hospital, Tongji Medical College, Huazhong University of Science and Technology, Wuhan, 430022 China; 5 Department of Neurology, The Friedman Brain Institute, Icahn School of Medicine at Mount Sinai, New York, New York, 10029 USA

## Abstract

Fluoxetine, one of the selective serotonin reuptake inhibitor (SSRI) antidepressants, has been thought to be effective for treating post-stroke depression (PSD). Recent work has shown that fluoxetine may exert an antidepressive effect through increasing the level of brain-derived neurotrophic factor (BDNF), but the underlying mechanism still remains unclear. In the present study, we successfully established the PSD model using male C57BL/6 J mice by photothrombosis of the left anterior cortex combined with isolatied-housing conditions. In the process, we confirmed that fluoxetine could improve the depression-like behaviors of PSD mice and upregulate the expression of BDNF in the hippocampus. However, depletion of BDNF by transfecting lentivirus-derived shBDNF in hippocampus suppressed the effect of fluoxetine. Furthermore, we demonstrated the epigenetic mechanisms involved in regulation of BDNF expression induced by fluoxetine. We found a statistically significant increase in DNA methylation at specific CpG sites (loci 2) of *Bdnf* promoter IV in the hippocampus of PSD mice. We also found that fluoxetine treatment could disassociate the MeCP2-CREB-*Bdnf* promoter IV complex via phosphorylation of MeCP2 at Ser421 by Protein Kinase A (PKA). Our research highlighted the importance of fluoxetine in regulating BDNF expression which could represent a potential strategy for preventing PSD.

## Introduction

Post-stroke depression (PSD) is a prevalent complex neuropsychiatric consequence of stroke, which can delay functional recovery and increase mortality^1^. At least one third of stroke survivors suffer from depression^2^. Specific pathophysiology of PSD is still under debate and lacking effective pharmacotherapy.

Both tricyclic antidepressants and selective serotonin reuptake inhibitors (SSRIs) were employed in initial studies of PSD treatment and both were effective. Due to the concerns regarding side-effects of tricyclic antidepressants, the following studies employed mostly SSRIs^1^. Fluoxetine, as one of the most commonly prescribed SSRI antidepressants, has been thought to be effective and safe for patients with PSD in light of some randomized controlled trials^3,4^. Brain-derived neurotrophic factor (BDNF), a member of the neurotrophin family, plays important roles in the proliferation, differentiation and survival of neurons in the central nervous system. Importantly, BDNF has been repeatedly implicated in the pathology of depression and antidepressant treatment^5,6^. Previous studies have shown that fluoxetine could increase BDNF levels in the hippocampus of depressive animal models^7,8^. In this study, we also found that chronic treatment with fluoxetine could upregulate BDNF levels and improve depressive like behaviors in PSD mice. This suggests that BDNF could attribute to pharmacological effects of fluoxetine in treating depression. However, the underlying mechanism by which fluoxetine up-regulates BDNF level is still unknown.

DNA methylation is one of the main epigenetic mechanisms to control *Bdnf* gene transcription^9^. It is known that the *Bdnf* gene comprises nine 5′ non-coding exons (I-IXa), each of which is linked to individual promoter regions, and a 3′ coding exon (IXb), which codes for the BDNF pre-protein amino acid sequence^10^. Considerable evidence suggests a crucial role for the *Bdnf* promoter methylation in patients with depression^11,12^. In this study, we found higher *Bdnf* promoter IV methylation status in the hippocampus of PSD mice. *Bdnf* promoter IV is a preferential target for epigenetic alterations, as it contains binding sites for cAMP-response element binding protein (CREB) and methyl-CpG-binding protein 2 (MeCP2), two vital transcriptional regulators known to mediate gene expression at the epigenetic level^13,14^. CREB can bind to the CRE element and induce BDNF expression. Under physiological condition, MeCP2 specifically combines with methylated *Bdnf* promoters to form a repressor complex, which could inhibit CREB binding to the CRE element and finally suppress *Bdnf* gene transcription^15^. Evidences show that phosphorylation of MeCP2 at serine-421 (pMeCP2) induces disassociation of CREB from the repressor complex^16,17^. It is reported that fluoxetine could activate a protein kinase A (PKA)-CREB signal pathway and then up-regulate phosphorylated CREB (pCREB) levels in depression cell model^18^. This raises the question as to whether fluoxetine can induce BDNF expression through affecting MeCP2 or CREB levels in PSD model.

The approaches take to establish a PSD experimental model in rodents have concerned psychosocial factors, biologic mechanisms and neuroanatomy^19,20^. In the present study, we employed ischemia in the left anterior cortical layers by photothrombosis combined with isolated-housing to establish a PSD mouse model. The major advantages of this model are that they possess persistent depression-like behaviors without motor impairments that could interfere with assessments of depression^20–22^. Since a single behavioral measure to assess the antidepressant effect is easily liable to misinterpretation, we combine three depression evaluation methods to better cover the whole spectrum of depressive-like behaviors in PSD mice^20^.

In this study, we conducted experiments on a PSD mice model, detected the depression by behavior tests, and determined the methylation of *Bdnf* promoter IV by mass spectrometry and the expression of BDNF, PKA, CREB, pCREB, MeCP2, pMeCP2 by Western blot assay. In addition, we further quantified the binding of pCREB to *Bdnf* promoter IV by chromatin immunoprecipitation assay (ChIP), as well as the interaction of CREB and MeCP2 by co-immunoprecipitation (Co-IP). In summary, we identified that the antidepressive effect of fluoxetine in PSD mice is mediated by inducing BDNF expression in the hippocampus via the activation of PKA, which is able to disassociate MeCP2-CREB-*Bdnf* promoter IV complex by epigenetic modification.

## Results

### Fluoxetine can improve the depression-like behaviors in PSD mice

Cortical ischemia induced by photothrombosis is verified in Figure S1. As shown in Figure S2a, before the experiment, all of the mice were subjected to the first behavioral tests (behavioral tests-1), including a forced swimming test (FST) and a sucrose preference test (SPT), then divided into control group without treatment (con), sham group with only rose bengal injection but without illumination and isolatied-housing (sham) and ischemia group with photothrombosis and isolatied-housing (ischemia group). The neurological functions of each mouse were evaluated before and at 1, 7, 14, 28 days and 2 months after ischemia using the Modified Neurological Severity Score (mNSS). Next, the second behavioral tests (behavioral tests-2) were carried out to confirm the mice with depressive mood. After this section, mice with ischemia were divided into a PSD group and a post stroke non-depression (PSND) group.

As shown in Figure S2b, mice recovered completely 2 months after ischemia, while mNSS had no significant difference between PSD and PSND mice (n = 18 mice per group, P > 0.05, two-way ANOVA, Figure S2b). As shown in Figure S2c–e, PSD mice had more severe depressive-like behaviors than the others. In the FST test, PSD mice showed more immobility time (n = 18 mice per group, P < 0.01, one-way ANOVA followed by Newman-Keuls multiple comparisons, Figure S2c) and less climbing time (n = 18 mice per group, P < 0.01, one-way ANOVA followed by Newman-Keuls multiple comparisons, Figure S2d). In the SPT test, sucrose preference of PSD mice decreased significantly (n = 18 mice per group, P < 0.01, one-way ANOVA followed by Newman-Keuls multiple comparisons, Figure S2e). In addition, we found that our PSD mice had anxiety phenotype which was the same as other PSD mouse models^22^. In the open-field test (OFT), PSD mice spent less time in the center of the open-field (n = 18 per group, P < 0.01, one-way ANOVA followed by Newman-Keuls multiple comparisons, Figure S2f) and had less entries into the center of the open-field (n = 18 per group, P < 0.01, one-way ANOVA followed by Newman-Keuls multiple comparisons, Figure S2e).

**Figure 1:**
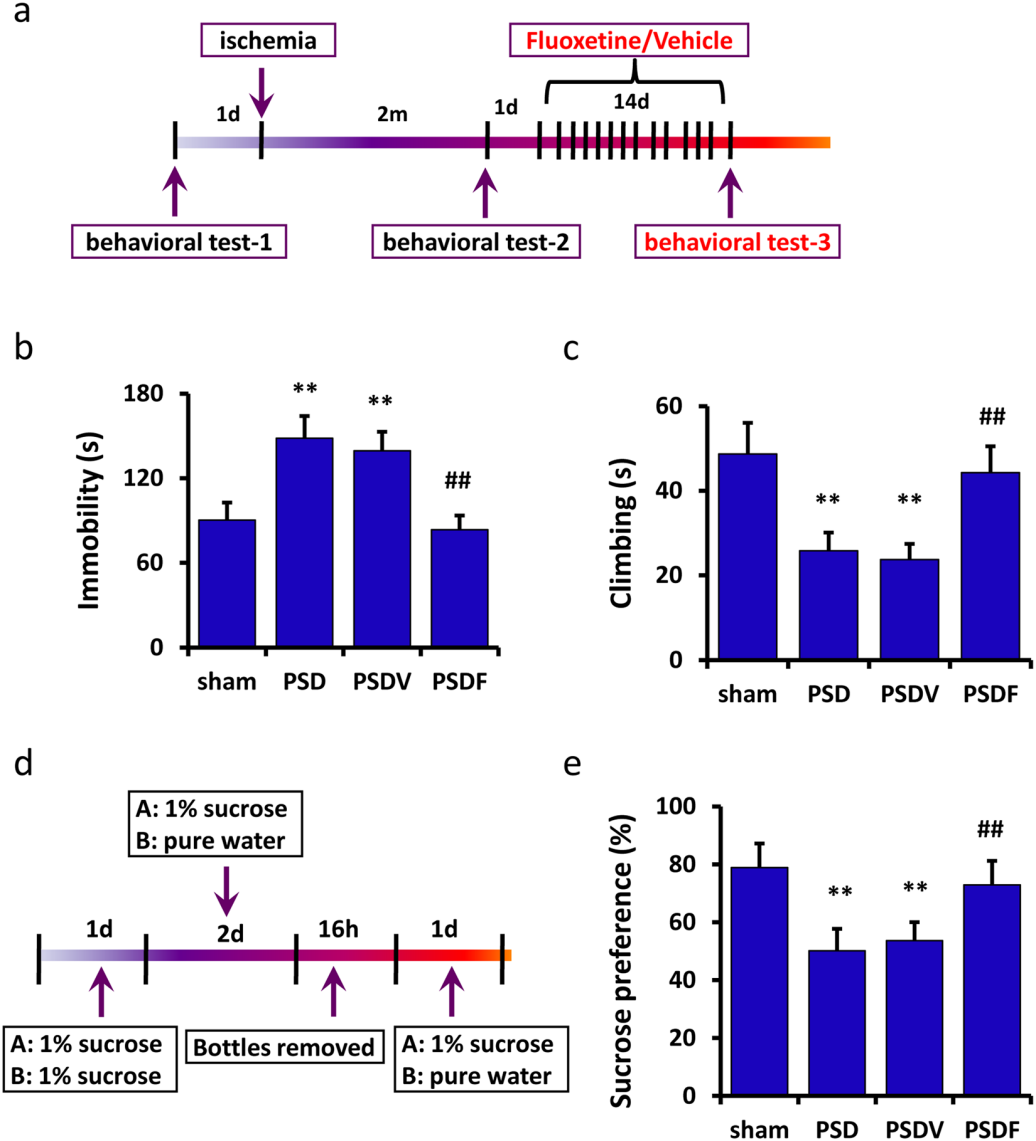
Fluoxetine can improve the depressive-like behaviors of PSD mice. (**a**) An illustration shows the experimental schedule. (**b**,**c**) In the forced swimming test, fluoxetine treatment was found to decrease the immobility time (**b**) and increase the climbing time (**c**) of the PSD mice (n = 8 per group, one-way ANOVA followed by Newman-Keuls multiple comparisons). (**d**) An illustration shows the experimental schedule of sucrose preference test. (**e**) In the sucrose preference test, fluoxetine treatment was found to increase the sucrose preference of PSD mice (n = 8 per group, one-way ANOVA followed by Newman-Keuls multiple comparisons). **P < 0.01 vs. sham group; ^##^P < 0.01 vs. PSDV group. Data are presented as the mean ± SEM. PSD: post stroke depression group; PSDV: PSD with vehicle injection group; PSDF: PSD with fluoxetine injection group.

Next, we administered fluoxetine (PSDF group) or vehicle (PSDV group) to PSD mice for 14 days (20 mg/kg, *i.p*., once per day), after which the third behavioral tests (behavioral tests-3) were carried out (Fig. 1a). As shown in Fig. 1b–e, the depressive behaviors in PSDF mice were improved significantly compared to the PSDV mice. The FST showed a significant decrease in the immobility time (n = 8 mice per group, P < 0.01, one-way ANOVA followed by Newman-Keuls multiple comparisons, Fig. 1b) and an increase in the climbing time (n = 8 mice per group, P < 0.01, one-way ANOVA followed by Newman-Keuls multiple comparisons, Fig. 1c) of PSDF mice as compared to the PSDV mice. In the SPT test, sucrose preference increased more significantly in the PSDF than the PSDV mice (n = 8 mice per group, P < 0.01, one-way ANOVA followed by Newman-Keuls multiple comparisons, Fig. 1d,e).

### Fluoxetine regulated BDNF expression in the hippocampus of PSD mice

Several studies have found that both depressed patients and mice models experience a great reduction of BDNF expression in serum and the brain^23^. To determine the possible role of BDNF in the PSD mice and the potential antidepressive effect of fluoxetine, the expression levels of BDNF in the hippocampus were investigated using the RT-PCR and Western blot.

**Figure 2:**
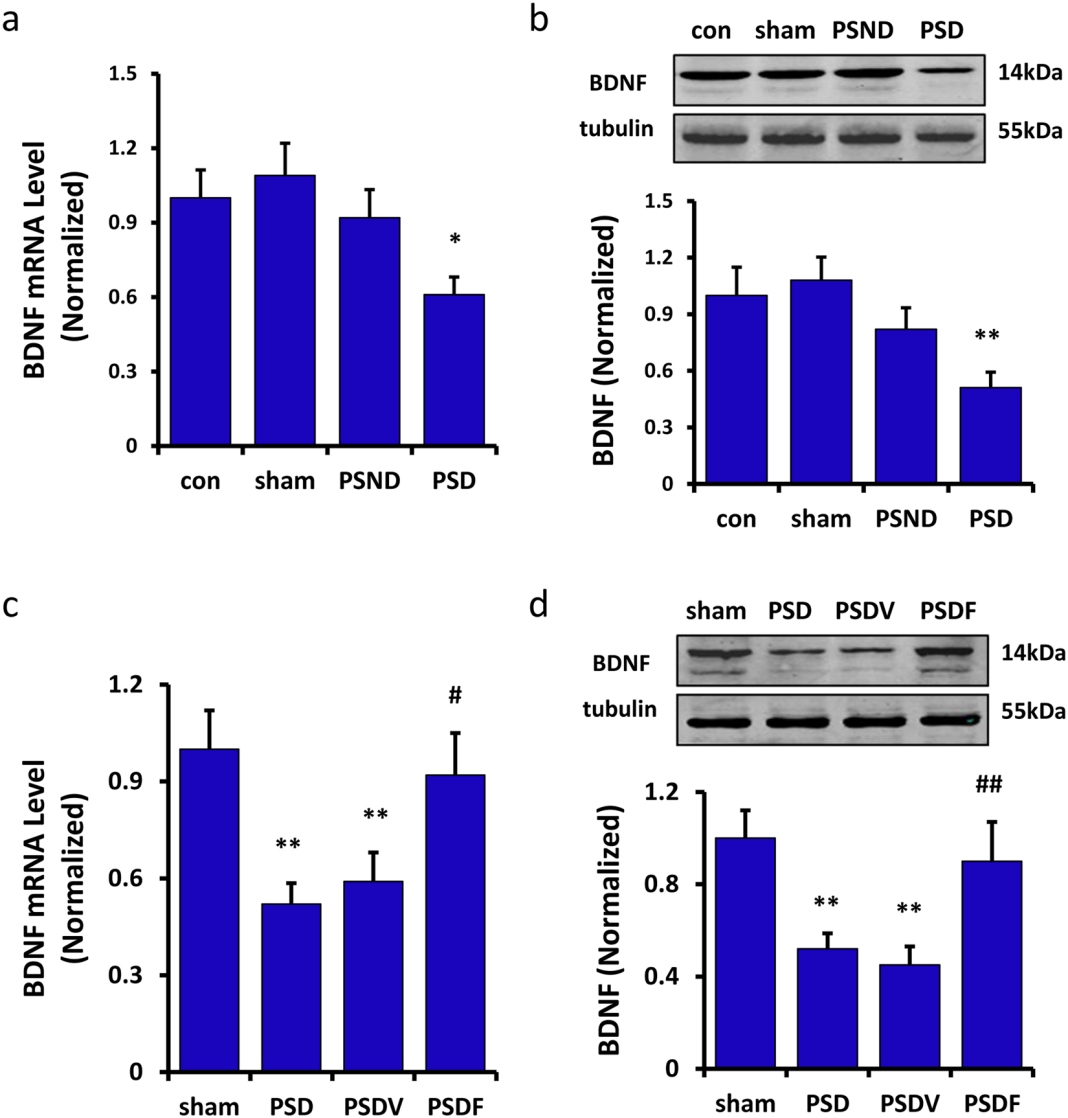
Fluoxetine was found to retrieve BDNF levels in the hippocampus of the PSD mice. (**a**,**b**) Total BDNF mRNA and protein levels in the hippocampus of the PSD mice were decreased significantly by RT-PCR (**a**) and Western blot (**b**) analysis (n = 6 per group, one-way ANOVA followed by Newman-Keuls multiple comparisons). (**c**,**d**) Fluoxetine treatment was found to increase the BDNF mRNA (**c**) and protein (**d**) expression of the PSD mice (n = 6 per group, one-way ANOVA followed by Newman-Keuls multiple comparisons). Full-length blots/gels are presented in Supplementary Figure 5. *P < 0.05, **P < 0.01 vs. sham group; ^#^P < 0.05, ^##^P < 0.01 vs. PSDV group. Data are presented as the mean ± SEM. PSD: post stroke depression group; PSND: post stroke with non-depression group; PSDV: PSD with vehicle injection group; PSDF: PSD with fluoxetine injection group.

Total BDNF mRNA and protein expression in the hippocampus of PSD mice were decreased significantly when compared with the sham group (RT-PCR: n = 6 mice per group, P < 0.05, one-way ANOVA followed by Newman-Keuls multiple comparisons, Fig. 2a; Western blot: n = 6 mice per group, P < 0.01, one-way ANOVA followed by Newman-Keuls multiple comparisons, Fig. 2b). Notably, the results also showed that the BDNF expression in the PSND group was decreased mildly, when compared with the sham group (RT-PCR: n = 6 mice per group, P > 0.05, Fig. 2a; Western blot: n = 6 per group, P > 0.05, Fig. 2b). We also detected the expression of TrkB and pTrkB finding no significant difference among these groups (n = 6 mice per group, P > 0.05, one-way ANOVA, Figure S3a–c). Chronic treatment with fluoxetine significantly ameliorated the reduction in BDNF expression when compared with the PSDV group (RT-PCR: n = 6 mice per group, P < 0.05, Fig. 2c; Western blot: n = 6 mice per group, P < 0.01, Fig. 2d, one-way ANOVA followed by Newman-Keuls multiple comparisons). These results suggested that the antidepressant mechanism of fluoxetine which may be related to the changes of BDNF expression in the hippocampus. But the expression of TrkB and pTrkB in the hippocampus had no significant difference (n = 6 mice per group, P > 0.05, one-way ANOVA, Figure S3d–f).

### Fluoxetine improves the depressive-like behaviors of PSD mice through up-regulating BDNF expression

**Figure 3:**
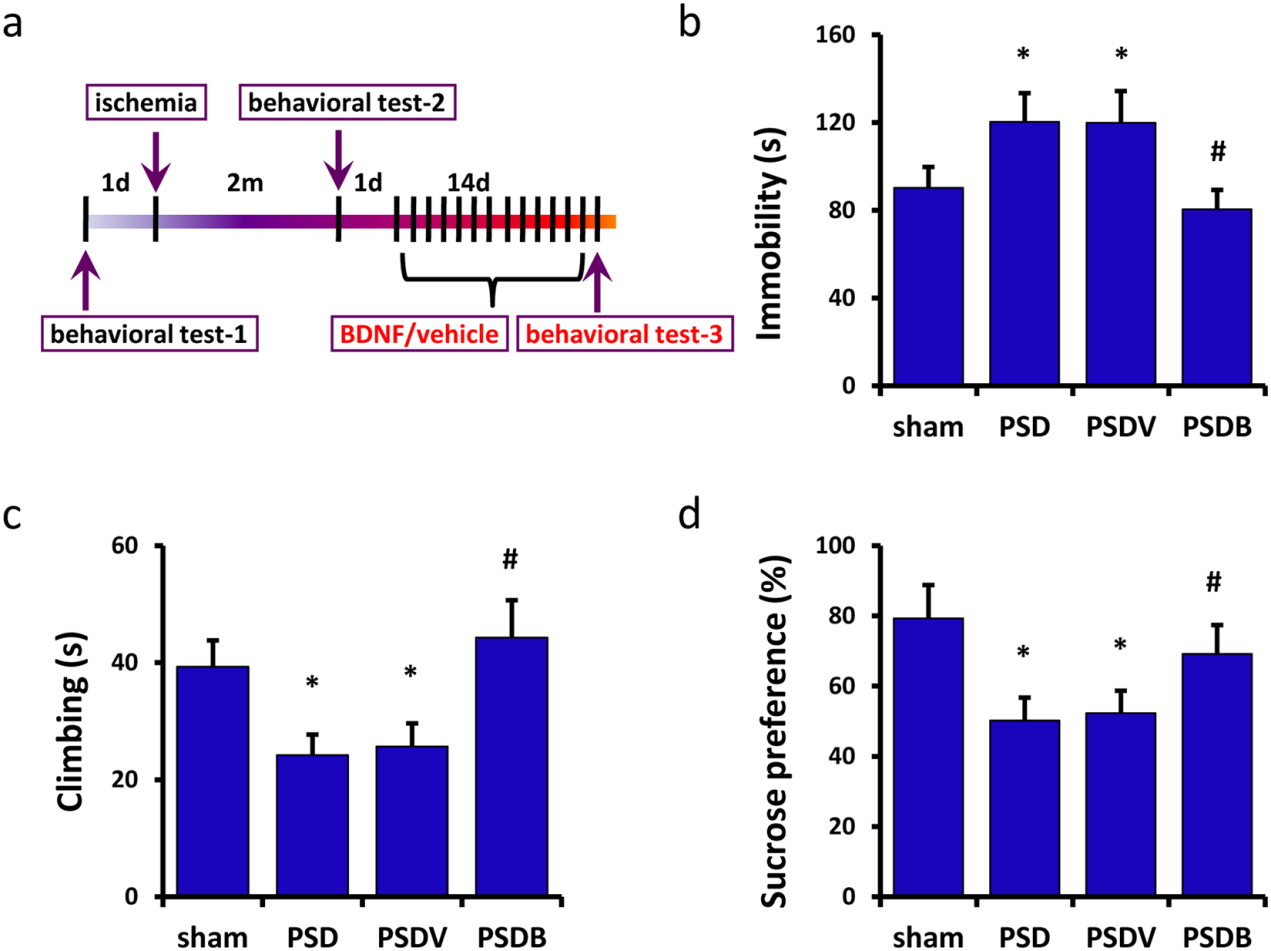
BDNF was found to improve the depressive-like behaviors of PSD mice. (**a**) An illustration shows the experimental schedule. (**b**,**c**) In the forced swimming test, BDNF treatment was found to decrease the immobility time (**b**) and increase the climbing time (**c**) of the PSD mice (n = 8 per group, one-way ANOVA followed by Newman-Keuls multiple comparisons). (**d**) In the sucrose preference test, BDNF treatment was found to increase the sucrose preference of the PSD mice (n = 8 per group, one-way ANOVA followed by Newman-Keuls multiple comparisons). *P < 0.05 vs. sham group; ^#^P < 0.05 vs. PSDV group. Data are presented as mean ± SEM. PSD: post stroke depression group; PSDV: PSD with vehicle injection group; PSDB: PSD with BDNF injection group.

Next, we injected BDNF (PSDB group) or vehicle (PSDV group) into the lateral ventricle of the PSD mice for 14 days (2 μg/2 μl/mouse, once per day). Then we carried out the behavioral test-3 (Fig. 3a), finding that BDNF could improve depressive behaviors of PSD mice. In the FST, PSDB mice showed less immobility time (n = 8 mice per group, P < 0.05, one-way ANOVA followed by Newman-Keuls multiple comparisons, Fig. 3b) and more climbing time (n = 8 mice per group, P < 0.05, one-way ANOVA followed by Newman-Keuls multiple comparisons, Fig. 3c) when compared with the PSDV mice. In the SPT, PSDB mice consumed more sucrose (n = 8 mice per group, P < 0.05, one-way ANOVA followed by Newman-Keuls multiple comparisons, Fig. 3d) than the PSDV mice. We also tested the effect of BDNF injection on plasma corticosterone levels and body weight. The PSD mice had more increased plasma corticosterone levels and decreased body weight than the sham mice (n = 8 mice per group, P < 0.05, one-way ANOVA followed by Newman-Keuls multiple comparisons, Figure S4a,b), which was consistent with the findings of others^24^. But there was no significant difference between the PSDB and the PSDV mice (n = 8 mice per group, P > 0.05, one-way ANOVA followed by Newman-Keuls multiple comparisons, Figure S4a,b).

**Figure 4:**
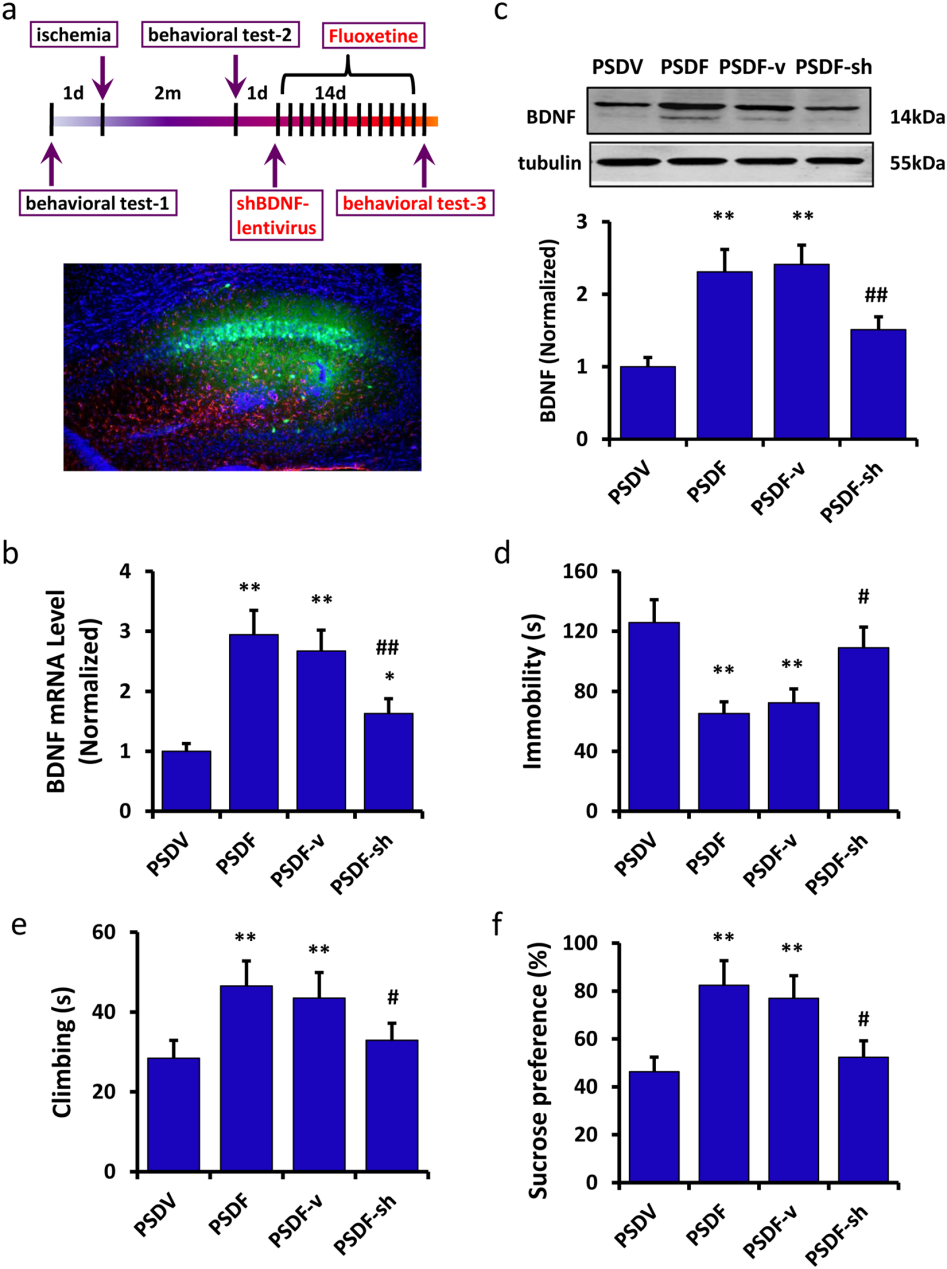
Fluoxetine was found to improve the depressive-like behaviors of PSD mice through up-regulating BDNF expression. (**a**) An illustration shows the experimental schedule (upper pannel) and representative image of the lentivirus-infected hippocampus area in the mouse brain (lower pannel). (**b**,**c**) Expression of BDNF mRNA (**b**) and protein (**c**) induced by fluoxetine treatment was remarkably decreased after transfection with BDNF-shRNA (n = 6 per group, one-way ANOVA followed by Newman-Keuls multiple comparisons). (**d**,**e**) In the forced swimming test, fluoxetine treatment induced a decrease in immobility time (**d**) and an increase in climbing time (**e**), which were reversed by BDNF silencing (n = 8 per group, one-way ANOVA followed by Newman-Keuls multiple comparisons). (**f**) In the sucrose preference test, the increase in sucrose consumption induced by fluoxetine treatment was reversed by BDNF silencing (n = 8 per group, one-way ANOVA followed by Newman-Keuls multiple comparisons). Full-length blots/gels are presented in Supplementary Figure 5. **P < 0.01 vs. PSDV group; ^#^P < 0.05, ^##^P < 0.01 vs. PSDF-v group. Data are presented as the mean ± SEM. PSDV: PSD with vehicle injection group; PSDF: PSD with fluoxetine injection group; PSDF-v: PSDF with non-targeting vector transfection group; PSDF-sh: PSDF with shBDNF-vector transfection group.

To further confirm that fluoxetine was able to improve the depressive-like behaviors of PSD mice by up-regulating BDNF expression, we used lentivirus-derived shBDNF (shBDNF1, shBDNF2, shBDNF3 and shBDNF4) to deplete the expression of BDNF. The transfection efficacy is shown in Figure S4c,d. As determined by qRT-PCR, shBDNF2 was the most efficacious (n = 6 per group, P < 0.01, one-way ANOVA followed by Newman-Keuls multiple comparisons, Figure S4d). Thus, shBDNF2 was chosen and renamed as shBDNF in the following experiments. Next, we injected shBDNF-lentivirus in the hippocampus of the PSD mice to silence BDNF (Fig. 4a) and then injected fluoxetine for 14 days (PSDF-sh group). The non-targeting vector was used as control (PSDF-v group). The BDNF mRNA (n = 6 mice per group, P < 0.01, one-way ANOVA followed by Newman-Keuls multiple comparisons, Fig. 4b) and protein expression (n = 6 mice per group, P < 0.01, one-way ANOVA followed by Newman-Keuls multiple comparisons, Fig. 4c), as induced by fluoxetine treatment was remarkably decreased after the silencing of BDNF. In the FST test, fluoxetine treatment induced a decrease in immobility time and an increase in climbing time; however, these effects were reversed by the silencing of BDNF (immobility time: n = 8 mice per group, P < 0.05, one-way ANOVA followed by Newman-Keuls multiple comparisons, Fig. 4d; climbing time: n = 8 mice per group, P < 0.05, one-way ANOVA followed by Newman-Keuls multiple comparisons, Fig. 4e). In the SPT test, the increase of sucrose preference induced by fluoxetine treatment was also reversed by the silencing of BDNF (n = 8 mice per group, P < 0.05, one-way ANOVA followed by Newman-Keuls multiple comparisons, Fig. 4f).

### Fluoxetine was unable to reverse DNA methylation at the *Bdnf* promoter IV after PSD

**Figure 5:**
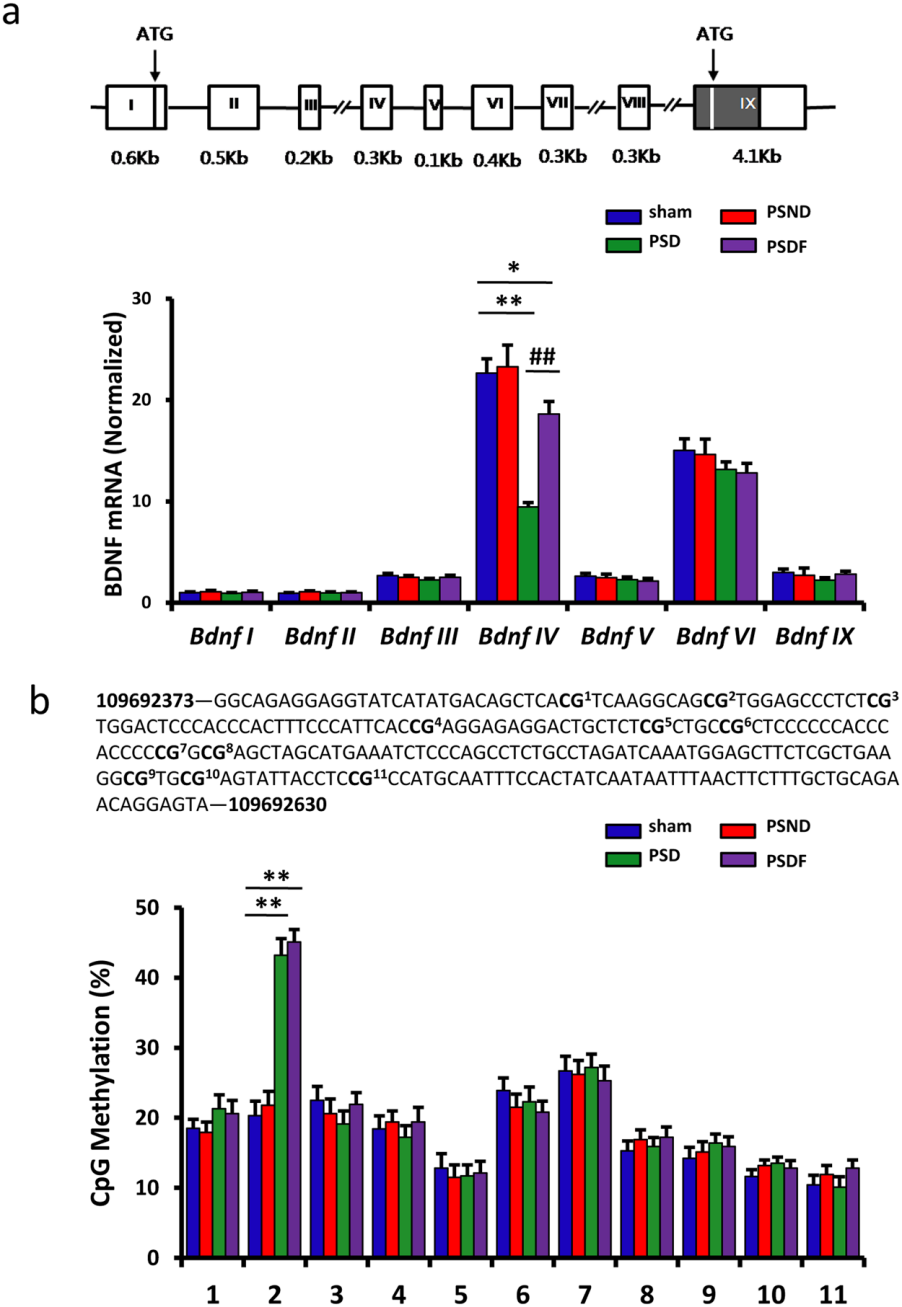
The treatment of fluoxetine cannot reverse the DNA methylation changes at the *Bdnf* promoter IV after PSD. (**a**) Quantification of the BDNF transcripts in the hippocampus among different groups by RT-PCR (n = 6 per group, one-way ANOVA followed by Newman-Keuls multiple comparisons). Illustration in the upper panel shows mice *Bdnf* exon gene structure (shown as boxes). (**b**) Quantification of the DNA methylation levels of *Bdnf* promoter IV in the hippocampus among different groups (n = 3 per group, one-way ANOVA followed by Newman-Keuls multiple comparisons). Illustration in the upper panel shows the mice *Bdnf* promoter IV gene structure.*P < 0.05, **P < 0.01 vs. sham group; ^##^P < 0.01 vs. PSD group. Data are presented as mean ± SEM. PSD: post stroke depression group; PSND: post stroke with non-depression group; PSDF: PSD with fluoxetine injection group.

To investigate the effect of fluoxetine on the BDNF expression in the hippocampus of the PSD mice, we measured 7 *Bdnf* transcripts (-I, -II, -III, -IV, -V, -VI, and -IX) expression in the hippocampus of sham, PSND, PSD and PSDF group mice using RT-PCR. *Bdnf*-VII and -VIII transcripts were excluded from the measurement due to the very low level of expression in the hippocampus. Compared with the sham and PSND groups, only the expression of BDNF-IV transcript was significantly down-regulated in the PSD group, which was reversible by the treatment of fluoxetine (PSDF group) (n = 6 mice per group, P < 0.01 for PSD group vs. sham group; P < 0.01 for PSDF group vs. PSD group; P < 0.05 for PSDF group vs. sham, one-way ANOVA followed by Newman-Keuls multiple comparisons, Fig. 5a).

Previous studies have indicated that the down-regulation of BDNF expression was associated with methylation on *Bdnf* promoters^25^. In particular, it has been suggested that the dynamic methylation of exon IV is an underlying mechanism which mediates BDNF expression during development and is susceptible to environmental insults. To explore the effects of fluoxetine on the regulation of methylation at the promoter of the *Bdnf* gene in the PSD mice, we carried out a quantitative analysis of the methylation of 11 CpG sites located in *Bdnf* promoter IV using the Sequenom MassARRAY platform (CapitalBio, Beijing, China). The hippocampus sample from the PSD and PSDF group mice showed a statistically significant increase in DNA methylation at specific CpG sites (loci 2) in *Bdnf* promoter IV compared with the sham group (n = 3 mice per group, P < 0.01, one-way ANOVA followed by Newman-Keuls multiple comparisons, Fig. 5b). Fluoxetine treatment was not sufficient to reduce the methylation level of this site compared with PSD mice (n = 3 mice per group, P > 0.05, Fig. 5b).

### Fluoxetine disassociated the MeCP2-CREB-*Bdnf* promoter IV complex via phosphorylating MeCP2 at Ser421

**Figure 6:**
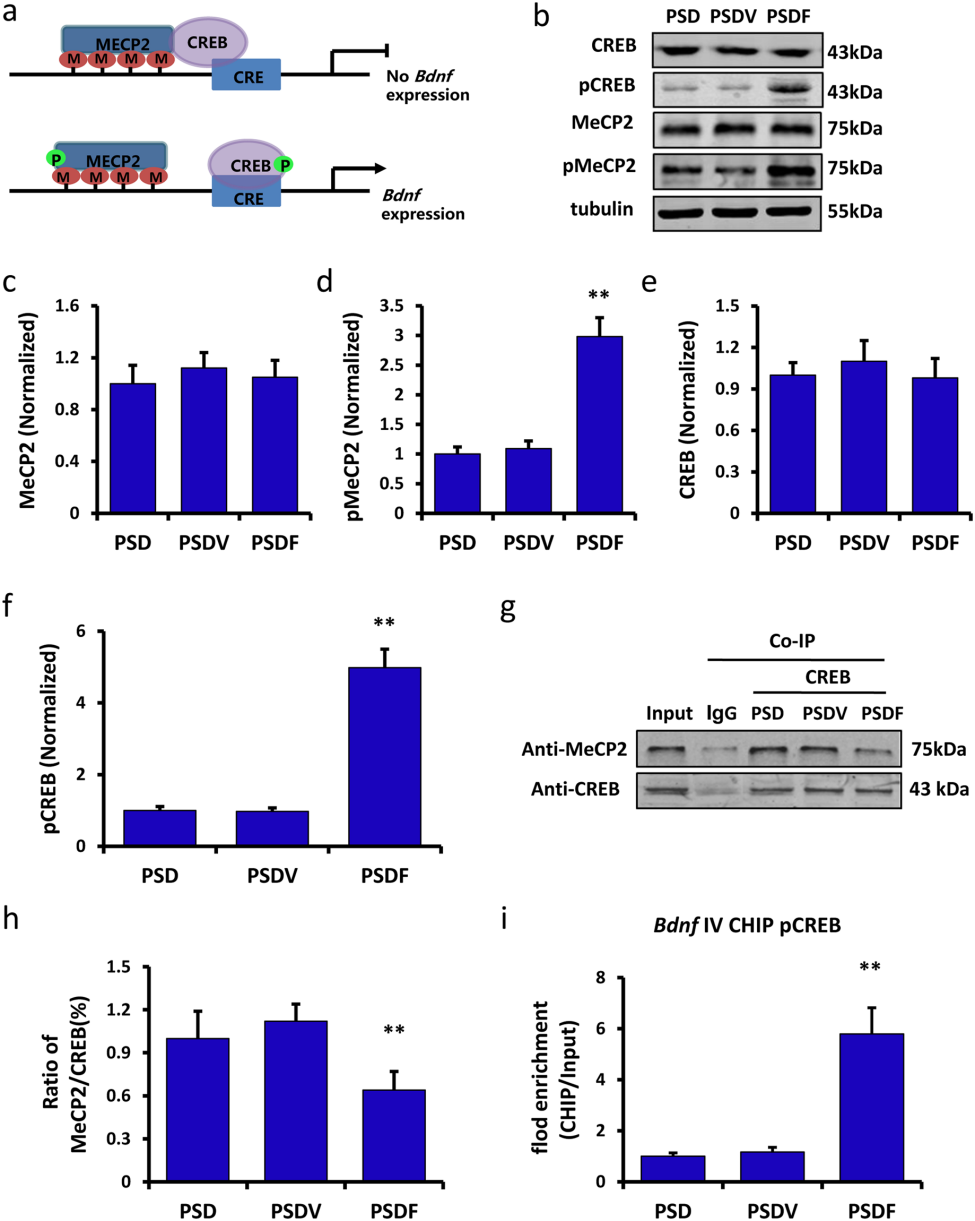
Fluoxetine was found to disassociate the MeCP2-CREB-*Bdnf* promoter IV complex via the phosphorylation of MeCP2. (**a**) Illustration shows the effect of MeCP2-CREB-*Bdnf* promoter IV complex on *Bdnf* gene expression. As shown in the upper panel, MeCP2 combined with methylated *Bdnf* promoters to form a repressor complex, which inhibited the CREB binding to the CRE and suppressed *Bdnf* gene transcription; As shown in the lower panel, the phosphorylated MeCP2 disassociated the CREB from this repressor complex, after which the CREB combined with the CRE and induced *Bdnf* gene transcription. (**b**) Immunoblot experiments show the protein levels of MeCP2, pMeCP2, CREB and pCREB among the PSD, PSDV and PSDF groups. (**c**–**f**) Quantification of the protein levels and the band intensities of MeCP2 (**c**), pMeCP2 (**d**), CREB (**e**) and pCREB (**f**) (n = 6 per group, one-way ANOVA followed by Newman-Keuls multiple comparisons). (**g**) Co-IP of 500 μg of proteins from the hippocampus with nonspecific IgG (IgG), anti-MeCP2 or anti-CREB. Input: 20 μg of protein from the extracts without IP was loaded. (**h**) Quantification of the Co-IP of the MeCP2/CREB ratio among the PSD, PSDV and PSDF groups (n = 6 per group, one-way ANOVA followed by Newman-Keuls multiple comparisons). (**i**) ChIP-qPCR assay of *Bdnf* promoter IV combined with pCREB among PSD, PSDV and PSDF groups (n = 6 per group, one-way ANOVA followed by Newman-Keuls multiple comparisons). Full-length blots/gels are presented in Supplementary Figure 5. *P < 0.05, **P < 0.01 vs. PSDV group. Data are presented as the mean ± SEM. PSD: post stroke depression group; PSDV: PSD with vehicle injection group; PSDF: PSD with fluoxetine injection group.

CREB, an important transcription factor, combines with CRE in order to regulate *Bdnf* gene transcription. The transcription level could be down-regulated via MeCP2, which is able to combine with methylated *Bdnf* promoters to form a repressor complex. This complex can bind CREB and suppress CREB binding to CRE, resulting in the inhibition of *Bdnf* gene transcription (Fig. 6a, upper panel)^15,26^. The phosphorylation of MeCP2 at Ser421 was reported to dissociate CREB from MeCP2-*Bdnf* promoter repressor complex (Fig. 6a, lower panel)^16,17^. To demonstrate the possibility that disassociation of the MeCP2-CREB-*Bdnf* promoter IV complex can contribute to the antidepressive effect of fluoxetine, we performed Western blot, Co-IP and ChIP-qPCR assays among different groups.

The PSD mice were treated with either vehicle or fluoxetine for 14 days (PSDV group and PSDF group). Then MeCP2, pMeCP2, CREB and pCREB protein levels in the hippocampus were detected by Western blot assay. No significant change was observed at the MeCP2 level in the hippocampus among the PSD, PSDV and PSDF groups (n = 6 mice per group, P > 0.05, one-way ANOVA, Fig. 6b,c). However, the pMeCP2 level was robustly increased in the PSDF group compared with the PSDV group (n = 6 mice per group, P < 0.01, one-way ANOVA followed by Newman-Keuls multiple comparisons, Fig. 6b,d). Similarly, the CREB level in the hippocampus exhibited no significant change (n = 6 mice per group, P > 0.05, one-way ANOVA, Fig. 6b,e), while the pCREB level was increased dramatically in the PSDF group compared with the PSDV group (n = 6 mice per group, P < 0.01, one-way ANOVA followed by Newman-Keuls multiple comparisons, Fig. 6b,f). Although the MeCP2 and CREB levels experienced no changes, the Co-IP analysis showed that the interaction of CREB and MeCP2 was decreased in the PSDF group (n = 6 mice per group, P < 0.01, one-way ANOVA followed by Newman-Keuls multiple comparisons, Fig. 6g,h). Meanwhile, the mRNA level of *Bdnf* promoter IV, which combined with pCREB in the hippocampus, was substantially increased in the PSDF group by ChIP-qPCR assay, compared with the PSDV group (n = 6 mice per group, P < 0.01, one-way ANOVA followed by Newman-Keuls multiple comparisons, Fig. 6i). These results suggest that fluoxetine treatment can phosphorylate MeCP2 and CREB in order to dissociate CREB from the MeCP2-CREB-*Bdnf* promoter IV repressor complex, and finally induce BDNF gene transcription.

### The phosphorylation of MeCP2 and CREB by fluoxetine treatment was reliant on PKA expression

**Figure 7:**
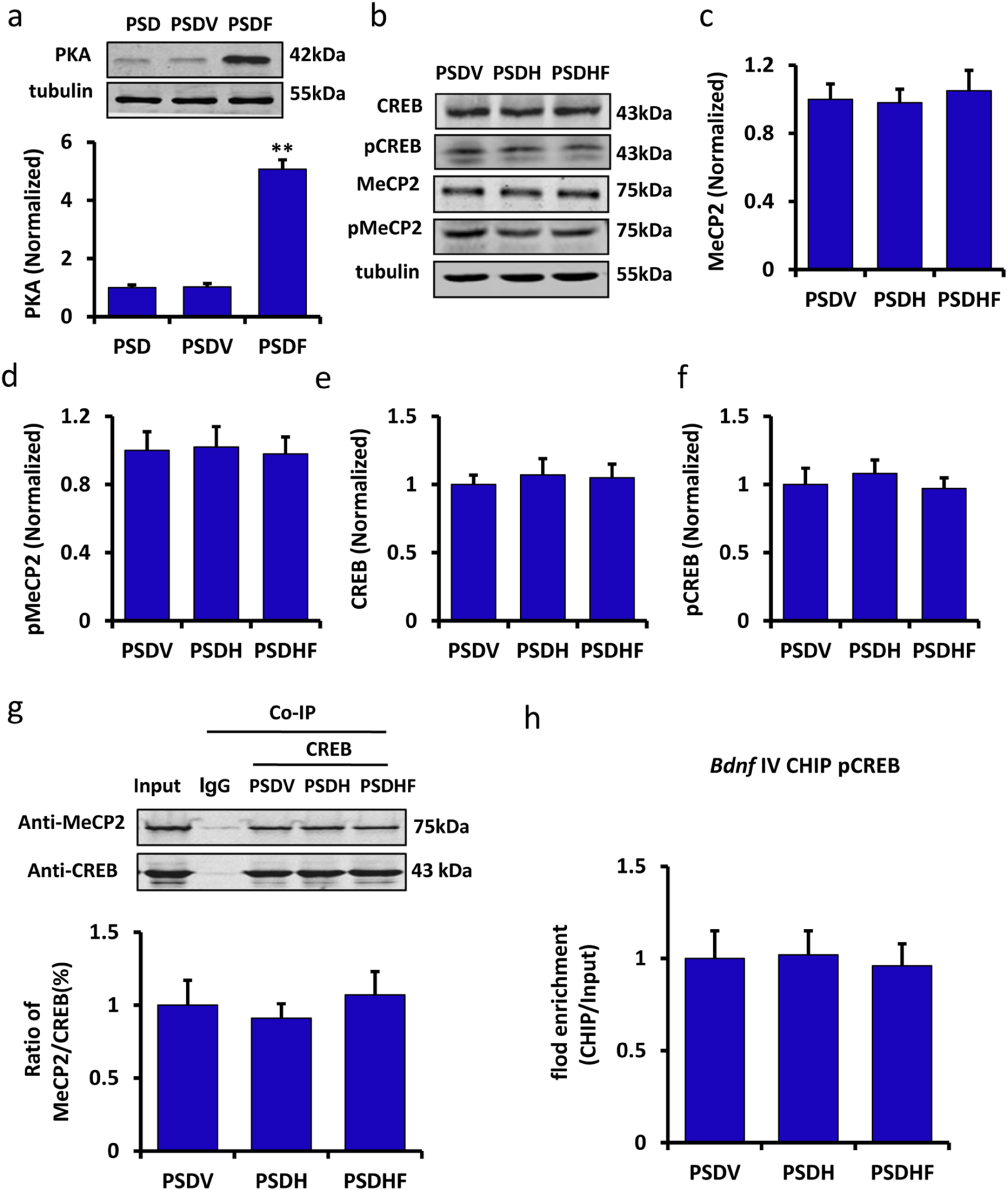
The phosphorylation of MeCP2 and CREB after fluoxetine treatment relied on PKA expression. (**a**) Immunoblot experiments show the protein levels of PKA among the PSD, PSDV and PSDF groups (n = 6 per group, one-way ANOVA followed by Newman-Keuls multiple comparisons). The lower panel shows the quantification of the protein levels of PKA. (**b**) Immunoblot experiments show the protein levels of MeCP2, pMeCP2, CREB and pCREB among the PSDV, PSDH and PSDHF group. (**c**–**f**) Quantification of the protein levels and band intensities of MeCP2 (c), pMeCP2 (**d**), CREB (**e**) and pCREB (**f**) (n = 6 per group, one-way ANOVA). (**g**) Co-IP of 500 μg of proteins from the hippocampus with nonspecific IgG (IgG), anti-MeCP2 or anti-CREB. Input: 20 μg of protein from the extracts without IP was loaded. The lower panel shows the quantification of the Co-IP of MeCP2/CREB ratio among the PSDV, PSDH and PSDHF groups (n = 6 per group, one-way ANOVA). (**h**) ChIP-qPCR assay of *Bdnf* promoter IV combined with pCREB among the PSDV, PSDH and PSDHF groups (n = 6 per group, one-way ANOVA). Full-length blots/gels are presented in Supplementary Figure 5. **P < 0.01 vs. PSDV group. Data are presented as the mean ± SEM. PSD: post stroke depression group; PSDV: PSD with vehicle injection group; PSDF: PSD with fluoxetine injection group; PSDH: PSD with PKA inhibitor H-89 injection group; PSDHF: PSD with H-89 and fluoxetine injection group.

It has been reported that PKA can phosphorylate CREB in the cell model of depression^18^. As such, we performed a Western blot analysis to test whether PKA could be modulated by fluoxetine treatment in the PSD mice. Our results showed that fluoxetine could significantly induce PKA expression in the PSDF group compared with the PSDV group (n = 6 mice per group, P < 0.01, one-way ANOVA followed by Newman-Keuls multiple comparisons, Fig. 7a).

Next, the PSD mice were treated with vehicle (PSDV group), PKA inhibitor H-89 (PSDH group), H-89 and fluoxetine together (PSDHF group) for 14 days. We then detected the expression of MeCP2, pMeCP2, CREB and pCREB in the hippocampus by Western blot assay. There was no significant change in any of the MeCP2, pMeCP2, CREB and pCREB proteins in the PSDV, PSDH and PSDHF groups (n = 6 mice per group, P > 0.05, one-way ANOVA, Fig. 7b–f). Meanwhile, we measured the combination of CREB and MeCP2 by Co-IP analysis (Fig. 7g), as well as the combination of *Bdnf* promoter IV and pCREB by ChIP-qPCR assay (Fig. 7h) in the hippocampus. No significant change in the PSDV, PSDH and PSDHF groups could be found (n = 6 mice per group, P > 0.05, one-way ANOVA, Fig. 7g,h). The above results suggested that the phosphorylation of MeCP2 and CREB, when induced by fluoxetine treatment is dependent on PKA activation.

## Discussion

While PSD is one of the leading causes of disability and mortality after ischemic stroke, effective strategies to minimize brain damage and improve recovery are still lacking. Accumulating evidence suggests that fluoxetine is effective for PSD models, which may be mediated by BDNF and neurogenesis^7,8^. In the present study, we demonstrated that, 1) fluoxetine was able to improve the depressive-like behaviors of the PSD mice through up-regulating the expression of BDNF in the hippocampus; 2) *Bdnf* promoter IV in the hippocampus was significantly methylated in the PSD mice, but fluoxetine treatment was unable to reverse the methylation level; 3) the up-regulation of BDNF expression after fluoxetine treatment relied on the disassociation of the MeCP2-CREB-*Bdnf* promoter IV complex, which was mediated via the phosphorylation of MeCP2 at Ser421; and 4) fluoxetine treatment was able to activate PKA in order to phosphorylate MeCP2 and CREB. Thus, our study provides new insights into the mechanisms of fluoxetine in PSD treatment. Epigenetic regulation of BDNF expression could be a promising target for developing potential therapy for PSD. In addition, consistent with other PSD mouse models, we found that our PSD mice had an anxiety phenotype during the OFT^22^. Further research is still needed to determine whether fluoxetine can alleviate the anxiety-like behaviors of stroke mice.

PSD can be effectively simulated in experimental rodents via a variety of approaches. For example, the middle cerebral artery occlusion (MCAO) model in combination with social isolation, which evokes stroke- and stress-induced depressive-like behavior phenotypes is conducive to observe the effects of antidepressant medication^21^. However, the mice suffering from MCAO surgery generally showed massive cortical and striatal ischemia, which usually causes permanent sensory-motor impairment that may interfere with assessments of depression behaviors. Recent studies support the existence of a positive relation between PSD and damage to left hemispheric lesions, including the medial prefrontal cortex^1,22,27^. Therefore, we adopt the left anterior cortex ischemia model induced by photothrombosis and combined with isolation-housing, in order to establish a PSD mouse model.

To validate the antidepressive effect of fluoxetine on the PSD mice, we followed optimal practice guidelines for testing mice behaviors, which include using multiple behavioral tests and that have construct and face validity, as well as outcomes that are objective and sensitive enough to detect long-term depression^20^. Additionally, to ensure the replicability of the results, we rigorously assessed the PSD phenotype of the mice and eliminated the ischemic mice without depression (PSND mice) in accordance with the FST. More importantly, we found that there was a significant difference for the PSD group (but not the PSND group) both in the behavioral tests and BDNF expression, compared with sham group, which further confirmed our view and that of others that BDNF is implicated in the pathogenesis of depression^6,28^.

BDNF has been repeatedly implicated in the pathogenesis of psychiatric and neurological disorders^29^. Exposure to different types of physical or social stress can induce dendritic atrophy and reduce neurogenesis in the hippocampus of rodent models by down-regulating BDNF levels^30^. Similar changes have been observed in the hippocampus of post-mortem brain tissues of patients with depression^31,32^. BDNF replacement therapy is currently being investigated in animal models and clinical studies, including Huntington’s disease, Alzheimer’s disease and depression^33^. Our results are consistent with those in a previous report, which showed that BDNF concentration was decreased in the PSD model^34–36^. BDNF also appears to be highly involved in hypothalamic-pituitary-adrenal (HPA) axis regulation, playing an important role in the pathological mechanisms of stress-induced mental diseases such as depressive illness. In this study, we detected the plasma corticosterone levels and body weight of PSD mice after injecting BDNF into the lateral ventricle. We found that BDNF injection did not change corticosterone levels and body weight. This suggests that BDNF can improve depressive behaviors of PSD mice regardless of the HPA axis. Naert, G. *et al*. also found that decreased BDNF levels did not induce change in the basal HPA axis activity, but involved the HPA axis’s adaptive response to stress^37^.

In addition, we found that chronic treatment with fluoxetine can improve the depressive behaviors by up-regulating BDNF levels. As we known, BDNF acts through the high-affinity TrkB receptor in order to activate the phosphorylation of TrkB. But, in our experiment, we found that although the BDNF expression in the hippocampus was increased significantly, the pTrkB had no significant difference after fluoxetine treatment. We thought that pTrkB may vary in different sub regions of the hippocampus, though we tested pTrkB expression in the whole hippocampus.

The synthesis of BDNF is influenced by epigenetic and genetic profiles. The epigenetic modifications include covalent modifications of DNA (methylation) and post-translational modifications of histone N-terminal tails (acetylation, methylation, phosphorylation and ubiquitinoylation), as well as non-transcriptional gene silencing mechanisms (micro-RNAs)^32^. DNA methylation is one of the most stable forms of epigenetic variability involved in regulating the transcription and function of selected genes in the adult mammalian nervous system^38,39^. Increased CpG methylation in promoter regions of the *Bdnf* gene is reportedly correlated with the decreased synthesis of BDNF protein in neurons^40^. Considerable evidence suggests a crucial role for BDNF promoter methylation in patients with neuropsychiatric disorders, such as schizophrenia^25,41^, bipolar disorder^42^, depression^11,12^ and anxiety^11,43^. The *Bdnf* gene comprises nine 5′ non-coding exons (I-IXa), each of which is linked to individual promoter regions, and a 3′ coding exon (IXb), which codes for the BDNF pre-protein amino acid sequence^10^. The alternative promoters, together with alternative splicing and polyadenylation can produce at least 18 distinct BDNF mRNAs, which remarkably encode identical initial BDNF protein product^10,44^. In the nine *Bdnf* promoters, promoter IV is the most responsive to depression and antidepressive effects, both *in vitro* and *in vivo* ^45^. We thus detected the role of fluoxetine in regulating *Bdnf* promoter IV methylation in PSD mice. We found that DNA methylation at specific CpG sites (loci 2) in *Bdnf* promoter IV increased significantly in the hippocampus of the PSD mice, but chronic treatment with fluoxetine did not reverse the methylation level of this genetic locus.

It is known that BDNF expression can also be regulated by the dynamic recruitment of CREB and MeCP2, which are two transcriptional regulators known to bind to the *Bdnf* exon IV promoter region in order to mediate epigenetic changes^13,14^. CREB is a common downstream target of antidepressants. The phosphorylation of Serine 133 residue induces the activation of CREB, which recruits other transcriptional coactivators to combine with the CRE element in order to induce *Bdnf* gene transcription^46^. The role of CREB in the antidepressive effect of fluoxetine on depression has been investigated both *in vitro* and *in vivo* ^18,47^. It has been reported that MeCP2 together with CREB and other transcription factors (for example, USF1/2, CaRF and MeF2) can bind with *Bdnf* promoter IV to form a repressor complex^26^, which in turn can inhibit the binding of CREB with CRE and ultimately decrease BDNF expression. When MeCP2 was phosphorylated, CREB dissociated from this repressor complex, before binding to the CRE element finally increasing *Bdnf* gene transcription^15^. In this study, we found that fluoxetine was able to increase the phosphorylation of CREB and MeCP2 through activating PKA in the hippocampus of PSD mice. Fluoxetine treatment was found to be able to dissociate CREB from MeCP2 and increase the combination of pCREB with *Bdnf* promoter IV in order to induce more *Bdnf* gene transcription.

In this study, we clarified the possible mechanism of fluoxetine in inducing BDNF expression in PSD mice, which can phosphorylate MeCP2 and CREB via the activation of PKA, before disassociating the MeCP2-CREB-*Bdnf* promoter IV complex. Based on this study and the majority of studies conducted so far, it appears that BDNF fulfills an important role in the pathogenesis of PSD. Thus, modulating the expression of BDNF could be a potential strategy for preventing PSD. In addition, consistent with other PSD mouse models, we found that our PSD mice had an anxiety phenotype during the OFT^22^. Further research is needed to determine whether fluoxetine can alleviate the anxiety-like behaviors of stroke mice.

## Materials and Methods

### Animals

Adult (3 months old) male C57BL/6 J mice were housed in plastic cages in an air-conditioned room at 24 °C in a 12 h light-dark cycle (light on at 8:00 am) with food and water available ad libitum. Mice were group-housed about 4 per cage before the experiment. Ischemic mouse was housed individually to mimic social isolation during the experiment. All experiments were carried out during the light cycle. All experimental procedures were carried out in compliance with relevant guidelines and regulations of the institutional committee of animal care and use. Care and experiments with mice were also approved by institutional guidelines of the Animal Care and Use Committee of Huazhong University of Science and Technology, Wuhan, China.

### Drugs

Fluoxetine hydrochloride (Sigma, St. Louis, MO, USA) was prepared in 0.9% sodium chloride (*i.p*., 20 mg/kg, once per day for 14 consecutive days). The PKA inhibitor H-89 (Sigma, St. Louis, MO, USA) was dissolved in 0.5% dimethyl sulfoxide (DMSO) and injected (*i.p*., 10 mg/kg) 30 min before fluoxetine or vehicle administration. The recombinant human BDNF (Sigma, St. Louis, MO, USA) at 2 μg/mouse was dissolved in 2 μl of 0.9% sodium chloride for lateral ventricle injection. Unilateral cannulas for infusion of BDNF were implanted into the lateral cerebral ventricle (injection site: anteroposterior (AP), −0.1 mm; mediolateral (ML), +1.0 mm; and dorsoventral (DV), −3.0 mm from the bregma).

### Lentivirus-derived transfection of BDNF-targeted shRNA

Lentivirus containing green fluorescent protein gene was commercially obtained, which was a package containing four BDNF-targeted shRNAs (shRNA1, shRNA2, shRNA3, shRNA4) or a non-targeting vector as a negative control (vehicle) (Hu6-MCS-CMV-EGFP, GV115, GENECHEM, China). The target regions of the shRNA candidates were designed according to Yoo *et al*.^48^:

BDNF shRNA1: 5′-GCGCCCATGAAAGAAGTAAAC-3′

BDNF shRNA2: 5′-GGTGATGCTCAGCAGTCAAGT-3′

BDNF shRNA3: 5′-GGAGCCTCCTCTACTCTTTCT-3′

BDNF shRNA4: 5′-GGTCACAGTCCTAGAGAAAGT-3′.

BDNF knockdown was attained by the lentivirus-derived transfection of BDNF-targeted shRNAs into primary cultured neurons according to instruction. The transfection efficacy of shRNAs was determined by fluorescence microscopy. The expression of BDNF after transfection was detected by qRT-PCR.

### Viral injection

Mice were anesthetized with 5% chloral hydrate and fixed on a stereotaxic frame. Lentivirus suspension containing 1 × 10^9^ TU/ml was injected into the hippocampus at a rate of 0.2 μl/min (total volume 2 μl). The stereotaxic coordinates of the injection site were as follows: AP, −1.8 mm; ML, −1.6 mm; DV, −1.70 mm from the bregma.

### Post-stroke depression model

Left cortical ischemia was induced photochemically, given that this region has been implicated in PSD. We modified the method introduced by Labat-gest *et al*.^49^. Before surgery, the animals were anesthetized with 5% chloral hydrate. The skin above the skull was incised and a fiber-optic bundle mounted on a cold light source (ϕ = 1.5 mm, wavelength 560 nm, 150 W, aperture B2, 2750 K, KL 1500 LCD, Schott, Germany), was placed in close contact with the left skull surface with a focus at 1.4 mm posterior to the bregma and 3 mm lateral to the midline (left anterior cortex). Photosensitive dye rose bengal (Sigma, St. Louis, MO, USA) was injected intraperitoneally (15 mg/ml dissolved in sterile saline solution, 150 mg/kg body weight). Then, 5 min after the injection, we started the focal illumination of the skull for 15 min to ensure that the photothrombotic lesion located in the left anterior cortical layers without adjacent area or hippocampus injury. Next, the incisions were sutured and the animals were housed individually to mimic social isolation. We used sham-operated animals as control, which were subjected to the same procedure except for light irradiation.

### Magnetic resonance imaging

The cortical infarction was verified by brain magnetic resonance imaging (MRI) examination, which was performed with a GE Signa HDxt 3.0 T scanner (GE, Fairfield, USA) equipped with a 3 inches animal coil (Chenguang, Shanghai, China). Quantitative T2 measurements were performed with the following acquisition parameters: FOV = 40 × 40 mm^2^, TR/TE = 3,040 ms/133 ms, image matrix = 192 × 160, NEX = 12, slice thickness 1.0 mm, space 0.2 mm, time: 6 min 09 s. The infarct volume was calculated according to MRI T2 weighted phase, while the lesion volume was quantified by the summation of areas of hyperintensity on each slice, multiplied by slice thickness.

### TTC staining

The cortical ischemia could also be verified by 2,3,5-triphenyltetrazolium chloride (TTC) staining. Twenty-four hours after ischemia, animals were deeply anesthetized and killed for TTC staining. The brain was removed rapidly and frozen at −20 °C for 30 min. Coronal slices (6 slices from each mouse) were made at 1mm from the frontal tips, and sections were immersed in 2% TTC (Sigma, CA, USA) at 37 °C for 20 min in the dark. The presence of infarctions was determined by examining the negative area of TTC staining.

### Evaluation of neurological deficits

The neurological functions of each mouse were evaluated prior to injury, 1, 7, 14 days, 1 and 2 months after injury by an experimenter blinded to the treatment status of the groups using the mNSS (Table S1), which included motor, sensory, reflex and balance tests (normal score 0; maximal deficit score 18)^50^.

### Behavioral tests

We performed the behavioral tests on three occasions according to the experiment design. The first occasion was 2 days before ischemic surgery in order to test the basic behavior of mice. The mice were then randomly allocated to either the control, sham or ischemic group. The second occasion was at 2 months after ischemic surgery. According to the results on these two occasions involving the FST, the ischemic mice were divided into PSD and PSND group. The PSD criterion was defined on the basis of the immobility time during the FST, which is the most widely used test for depression in rodents^51^. If the immobility time in the second test increased by over 50%, compared to the first test, these mice were allocated to the PSD group; otherwise, they were included in the PSND group. The PSD mice were randomly divided into different groups according to the experiment design and in light of the third behavioral tests at 14 days after drug or vehicle treatment. The behavioral tests were conducted by experimenter blinded to the treatment status of the mice.

#### Forced swimming test (FST)

The experimental device was purchased from Tai Meng Technology Co., Ltd (Chengdu, China). The FST was performed between 8:00 AM and 12:00 PM. Before testing, mice were placed inside a vertical cylinder (25 cm height × 10 cm diameter) containing 15 cm of water maintained at 23–25 °C for 15 min in order to adapt to the novel environment. On the next day, each mouse was placed inside the same vertical cylinder and left there for 6 min. The immobility time and climbing time were videotaped during the last 4 min of the 6-min test. Each mouse was judged to be immobile when it ceased to struggle and remained floating motionless in the water, making only those movements that were necessary to keep its head above the water as previously described^52^.

#### Sucrose preference test (SPT)

The SPT followed the published procedure with minor modifications^53^. Briefly, on the first day, individually housed mice were given two bottles of 1% sucrose to adapt to the sucrose solution. On the second day, mice were given a bottle of pure water and a second bottle with 1% sucrose. On the third day, the position of the two bottles was switched to eliminate potential side preferences. After 3 days of habituation, both bottles were removed for 16 h overnight. The water and sucrose bottles were then reintroduced in reversed left/right locations to the mice for 24 h. Consumption of water, sucrose and total liquid intake was measured at the end of the test. Sucrose preference was calculated according to the following equation: Sucrose Preference = Volume _Sucrose_/(Volume _Sucrose_ + Volume _Water_) × 100%

### Quantitative RT-PCR

Total RNA was isolated using the Trizol reagent (Invitrogen) from micro-dissected hippocampus and 5 µg was used to synthesize cDNA using M-MLV reverse transcriptase following the manufacturer’s protocol (Invitrogen). RT-PCR reactions were performed using SYBR^®^ Green Realtime Master Mix and ABI PRISM^®^ 7700 (Applied Biosystems). The primer sequences for the genes analyzed are summarized in Table S2. Each sample was run in duplicate and repeated twice. Threshold cycle values were used to calculate the fold change in the transcript levels by using the 2^−ΔΔCt^ method. The relative mRNA expression levels were normalized to the tubulin gene.

### Western blot

The protein samples (50 µg of protein) were prepared from micro-dissected hippocampus and separated by 12% SDS-PAGE gel. Proteins were transferred to nitrocellulose membranes using a Bio-Rad Miniprotein III System wet transfer unit for 1 h at 4 °C. Transfer membranes were then incubated with blocking solution (5% nonfat dried milk dissolved in TBST buffer, pH 7.5) for 1 h at room temperature, then incubated with anti-BDNF (1:1000, Abcam), anti-PKA(1:1000, Imagenex), anti-CREB (1:1000, Cell Signaling), anti-pCREB (Ser133, 1:1000, Cell Signaling), anti-MeCP2 (1:2000, Cell Signaling), anti-pMeCP2 (Ser421, 1:1000, Abgent), anti-TrkB (1:1000, Millipore) and anti-pTrkB (Tyr706, 1:1000, Cell Signaling) overnight at 4 °C. Membranes were incubated with secondary antibodies for 1 hat 4 °C. Signal detection was performed with an enhanced chemiluminescence kit (Amersham Biosciences) and quantitated by using the GS-710 Calibrated Image Densitometer (Bio-Rad).

### Chromatin Immunoprecipitation (ChIP)

ChIP assays were carried out using a kit from Millipore according to the manufacturer’s instructions. The tissues of the hippocampus were cut into small pieces then cross-linked with 1% formaldehyde, lysed and sonicated at different conditions to optimize the shearing of genomic DNA with an average size of 500 bp. 1% supernatant of the sheared samples were saved as an input control. Five microliters of supernatant was diluted and mixed with prepared Magna ChIP Protein A/G Magnetic Beads, which connected with the anti-pCREB (Cell Signaling), then incubated at 4 °C overnight with rotation. Immunoprecipitated complexes were collected, washed, eluted. Next, the sample, which was added to ChIP elution buffer and proteinase K was incubated at 65 °C for 2 h, then at 95 °C for 15 min to reverse cross-links and remove protein. The eluted DNA was used as a template for quantitative PCR analysis. The data were normalized to those of the input DNA. The primers used for *Bdnf* promoter IV were: forward, 5′-AAAGCATGCAATGCCCT-3′; reverse, 5′-GAGATTTCATGCTAGCTCGC-3′.

### Co-IP

The hippocampal tissue was dissected out and lysed by brief sonication in lysis buffer with a protease inhibitor. The clarified lysate (500 µg protein) was incubated with non-specific IgG (2 µg), polyclonal rabbit anti-MeCP2 antibody (2 µg, Cell Signaling) or anti-CREB antibody (2 µg, Cell Signaling) overnight at 4 °C. The protein A or G magnetic beads (GE Healthcare, Barrington, IL) were added to the IP reaction product in order to catch the immune complex at 4 °C for 3 h. The Immunoprecipitated complexes on the beads were washed three times with washing buffer. Immunoprecipitated samples or IgG control samples were washed with ice-cold lysis buffer and dissociated by heating for 5 min in the loading buffer, before being subjected to Western blot analysis using anti-CREB and anti-MeCP2 antibody, respectively.

### DNA methylation analysis

Genomic DNA was extracted from the hippocampus by using the Wizard® SV Genomic DNA Purification System according to the manufacturer’s instructions. DNA concentration and purity were determined based on the absorbance at 260 and 280 nm. A total of 1 µg of genomic DNA from each sample was bisulfite-treated using the Methylamp DNA Modification Kit (Epigentek, Farmingdale, NY, USA). Meanwhile, the Sequenom MassARRAY platform (CapitalBio, Beijing, China) was used for the quantitative analysis of BDNF methylation. This system employs matrix-assisted laser desorption/ionization time-of-flight (MALDI-TOF) mass spectrometry in combination with RNA base-specific cleavage. Primers for *Bdnf* promoter IV methylation analysis were designed using Methprimer (http://www.urogene.org/methprimer/) (forward, 5′-GGTAGAGGAGGTATTATATGATAGTTTA-3′; reverse, 5′-TACTCCTATTCTACAACAAAAAAATTAA-3′). Mass spectra were obtained using the MassARRAY Compact MALDI-TOF (Sequenom), while their methylation ratios were generated using the Epityper software (Version 1.0, Sequenom, San Diego, CA, USA).

### Statistical analysis

All variance values in the text and figure legends are represented as the mean ± SEM. Data were analyzed using the one-way ANOVA followed by Newman-Keuls comparisons. Statistical analyses were performed using SPSS 13.0. Statistically significant differences were defined as P < 0.05.

## Electronic supplementary material

supplementary information
