# Supplementary material for: Alleviative effects of fluoxetine on depressive-like behaviors by epigenetic regulation of BDNF gene transcription in mouse model of post-stroke depression
Source: Sci Rep. 2017 Nov 2;7:14926. doi: 10.1038/s41598-017-13929-5 (PMC5668242; doi:10.1038/s41598-017-13929-5)

Alleviative effects of fluoxetine on depressive-like behaviors by epigenetic regulation of BDNF gene transcription in mouse model of post-stroke depression

Hui-Juan Jin [1#], Lei Pei [2, 3#], Ya-Nan Li [1#], Hui Zheng [4], Shuai Yang [1], Yan Wan [1], Ling Mao [1], Yuan-Peng Xia [1], Quan-Wei He [1], Man Li [1], Zhen-Yu Yue[5], Bo Hu[1*]

[1]Department of Neurology, Union Hospital, Tongji Medical College, Huazhong University of Science and Technology, Wuhan, 430022, China

[2]Department of Neurobiology, School of Basic Medicine, Tongji Medical College, Huahzong University of Science and Technology, Wuhan, 430030, China

[3]The Institute for Brain Research (IBR), Collaborative Innovation Center for Brain Science, Huazhong University of Science and Technology, Wuhan, 430030, China

[4]Department of Radiology, Union Hospital, Tongji Medical College, Huazhong University of Science and Technology, Wuhan, 430022, China

[5]Department of Neurology, The Friedman Brain Institute, Icahn School of Medicine at Mount Sinai, New York, New York 10029, USA

Correspondence to Bo Hu, Department of Neurology, Union Hospital, Tongji Medical College, Huazhong University of Science and Technology, Wuhan, 430022, China

Tel: +86-13707114863; Fax: +86-27-85726028; E-mail: hubo@mail.hust.edu.cn

[#]The first three authors contributed equally to this work.

Table S1 The modified neurological severity score (mNSS)

**Motor tests (normal=0; maximum=6)**	
Raising mouse by tail (normal=0; maximum=3)	
Flexion of forelimb	1
Flexion of hindlimb	1
Head moved>10° to vertical axis within 30 s	1
Placing mouse on floor (normal=0; maximum=3)	
Normal walk	0
Inability to walk straight	1
Circling toward the paretic side	1
Falls down to paretic side	0
**Sensory tests (normal=0; maximum=2)**	
Placing test (visual and tactile test)	1
Propioceptive test	1
(deep sensation, pushing paw against table edge to stimulate limb muscles)	
**Beam balance tests (normal=0; maximum=6)**	
Balance with steady posture	0
Grasps side to beam	1
Hugs beam and 1 limb falls down from beam	2
Hugs beam and 2 limbs falls down from beam, or spins on beam(>60s)	3
Attempts to balance on beam but fails off (>40s)	4
Attempts to balance on beam but fails off (>20s)	5
Fail off; no attempts to balance or hang on the beam (<20s)	6
**Reflex absence and abnormal movements (normal=0; maximum=4)**	
Pinna reflex (head shaken when auditory meatus is touched)	1
Corneal reflex (eye blink when cornea is lightly touched with cotton)	1
Startle reflex (motor response to a brief noise from clapping hands)	1
Seizure, myoclonus, myodystony	1
**Maximum points**	**(18)**

One point is given for an absent reflex tested or for the animal's inability to perform a task: 1-6 mild injury, 7-12 moderate injury, 13-18 severe injury

Table S2 RT-PCR Primers of *Bdnf* transcripts

***BDNF* transcript**	**F/R**	**Primer sequence**
I	F	AGTTGCTTTGTCTTCTGTAGTCGC
	R	CATTCACGCTCTCCACAGTCCC
II	F	GAGCAGAGTCCATTCAGCACCTTG
	R	CATTCACGCTCTCCACAGTCCC
III	F	GAGAGTTCCGGGTGCTGGCTTGGA
	R	CATTCACGCTCTCCACAGTCCC
IV	F	GAGTACATATCGGCCACCAAAGAC
	R	CATTCACGCTCTCCACAGTCCC
V	F	CCGCTGGCTGGCTGTCGCACGGTTC
	R	CATTCACGCTCTCCACAGTCCC
VI	F	TCTGCGGAACTCCAGGACAGCCTC
	R	CATTCACGCTCTCCACAGTCCC
IX	F	GCAGCTGGAGTGGATCAGTAA
	R	CATTCACGCTCTCCACAGTCCC
Total	F	CAGGTGAGAAGAGTGATGACC
	R	ATTCACGCTCTCCAGAGTCCC
tubulin	F	CAGGCCGGACAGTGTGGCAAC
	R	GGCTTCATTATAGTACACAGAGATTCG

Figure S1 Representative images show the area sizes of left anterior cortex infarct in photothrombosis ischemic mice. (a) Macroscopical view of photothrombotic lesion. (b) Hematoxylin eosin staining. (c) TTC staining. (d) T2-weighted MR images. (e) Quantification of infarct volumes.

Figure S2 The depression-like behaviors of PSD mice. (a) An illustration shows the experimental schedule. (b) The mNSS scores (n=18 mice/group, two way ANOVA followed by Newman-Keuls multiple comparisons). (c, d) In the forced swimming test, PSD mice had more immobility time (c) and less climbing time (d) (n=18 mice/group, one way ANOVA followed by Newman-Keuls multiple comparisons). (e) In the sucrose preference test, sucrose preference of PSD mice decreased significantly (n=18 mice/group, one way ANOVA followed by Newman-Keuls multiple comparisons). (f, g) In the open-field test, PSD mice spent less time in (f) and less entries into the center zone of the open-field (g) (n=18 mice/group, one way ANOVA followed by Newman-Keuls multiple comparisons). $^{**}P < 0.01$ vs. sham group. Data are presented as mean ± SEM. PSD: post stroke depression group; PSND: post stroke with non-depression group.

Figure S3 Fluoxetine treatment did not affect TrkB expression and phosphorylation in the hippocampus. (a) Immunoblots experiments show the protein levels of TrkB and pTrkB among control, sham, PSND and PSD group. (b-c) Quantification of the protein levels, band intensities of TrkB (b) and pTrkB (c) (n=6 mice/group, one way ANOVA). (d) Immunoblots experiments show the protein levels of TrkB and pTrkB among PSD, PSDV and PSDF group. (e-f) Quantification of the protein levels, band intensities of TrkB (e) and pTrkB (f) (n=6 mice/group, one way ANOVA). Full-length blots/gels are presented in Supplementary Fig. 5. Data are presented as mean ± SEM. PSD: post stroke depression group; PSND: post stroke with non-depression group; PSDV: PSD with vehicle injection group; PSDF: PSD with fluoxetine injection group.

Figure S4 (a) BDNF injection did not affect the plasma corticosterone levels of PSD mice (n=8 mice per group, one-way ANOVA followed by Newman-Keuls multiple comparisons). (b) BDNF injection did not affect the body weight of PSD mice (n=8 mice per group, one-way ANOVA followed by Newman-Keuls multiple comparisons). (c) Transfection efficiency of lentivirus-derived shRNA into primay neuron. (d) RT-PCR showed that BDNF expression in primay neuron was significantly down-regulated after transfection with lentivirus-derived shRNA (n=6 or 7/group, one way ANOVA followed by Newman-Keuls multiple comparisons). *P < 0.05; **P < 0.01 vs. sham group or vehicle group.

Figure S5 Full-length blots/gels.

Supplementary Methods

Open-field test (OFT)

The open-field consisted of an opaque plastic box ($50\times50\times30$ cm^3). The field was subdivided into a central sector (a central square 30×30 cm^2) and a peripheral sector (corresponding to the remaining portion of the field). The animals were placed individually into the center of an open-field and allowed to explore for 10 min under dim light. Their behaviors were tracked by a video tracking system (Tai Meng Technology Co., Ltd, Chengdu, China). The time in and the entries into the central zone were measured, and the total travel distance described as locomotor activity was analyzed. The open-field arena was thoroughly cleaned with 70% ethanol between each test.

Determination of Corticosterone Concentrations in Plasma

Mice were anesthetized 14 day after the BDNF injection. Blood from each mouse was obtained through the retro-orbital plexus into a centrifuge tube containing 10 μl of heparin solution. Plasma was collected by centrifugation at $3000\times g$ for 20 min at 4 °C and then kept at −80 ℃ until examination. The levels of corticosterone were analyzed using a commercially available ELISA kit (ADI-900-097, Enzo Life Sciences, USA) on the basis of the manufacturer's protocol.

Bodyweight Measurement

Each mouse's bodyweight was measured 14 day after the BDNF injection using a digital analytical balance (Tai Meng Technology Co., Ltd, Chengdu, China). The average body weight (g) for each mouse was calculated from 3 repeated measurements, and the mean value of body weight was compared among the four groups.

Figure S1

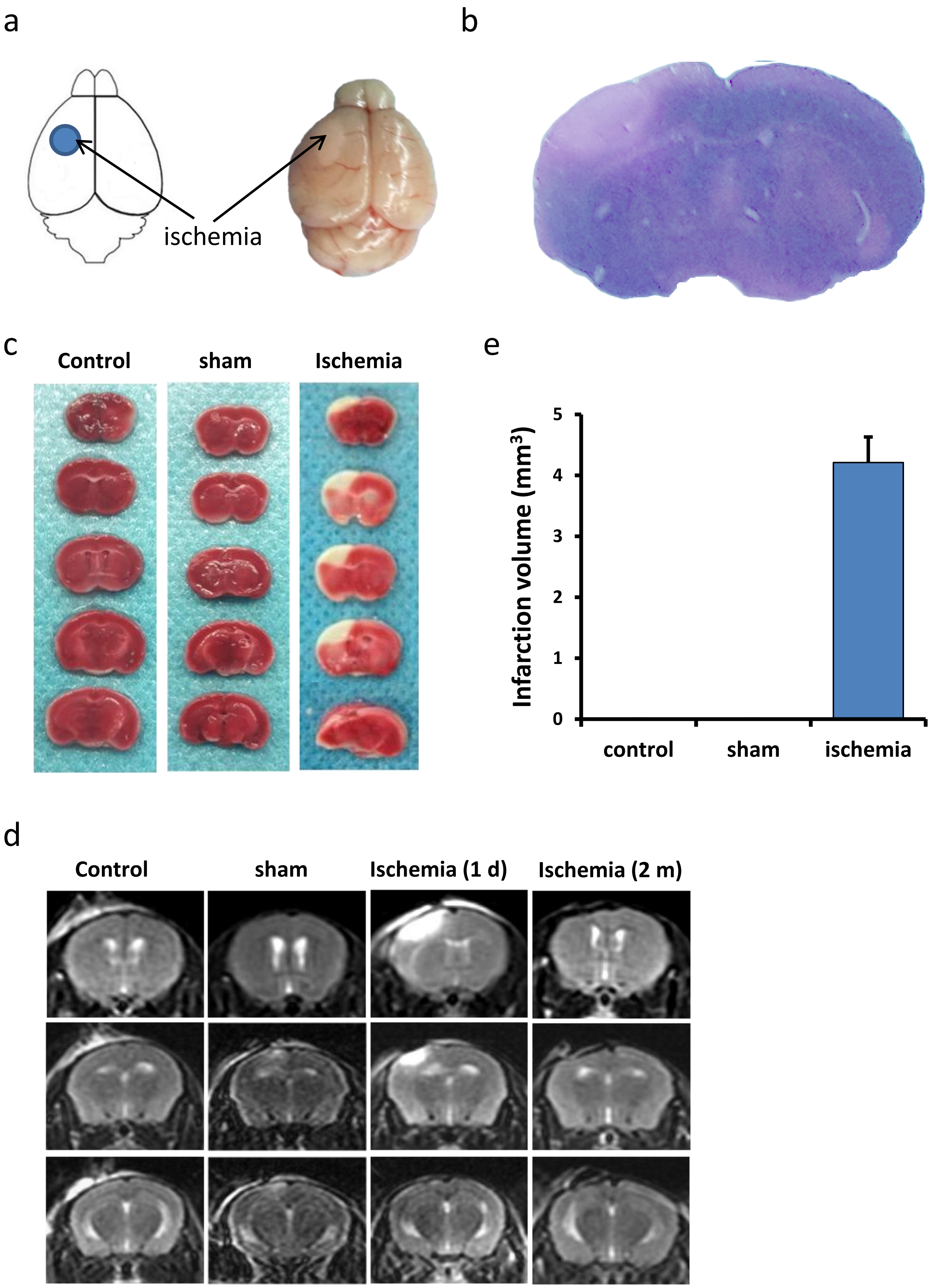
a
ischemia
b
c
Control
sham
Ischemia
e
Infarction volume (mm3)
5
4
3
2
1
0
control
sham
ischemia
d
Control
sham
Ischemia (1 d)
Ischemia (2 m)

Figure S2

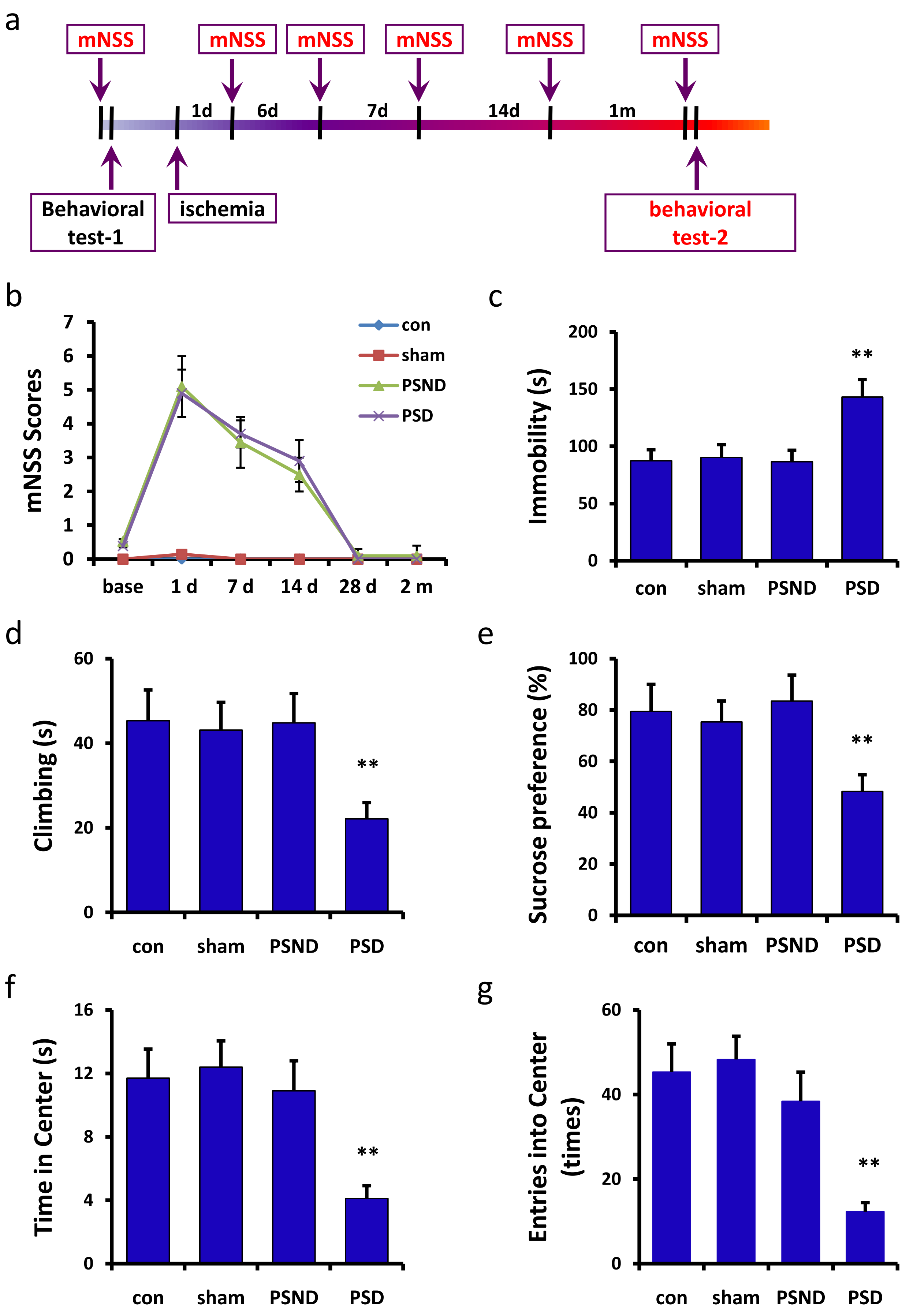
a
mNSS
mNSS
mNSS
mNSS
mNSS
mNSS
1d
6d
7d
14d
1m
Behavioral test-1
ischemia
behavioral test-2
b
mNSS Scores
7
6
5
4
3
2
1
0
base
1 d
7 d
14 d
28 d
2 m
con
sham
PSND
PSD
c
Immobility (s)
200
150
100
50
0
**
con
sham
PSND
PSD
d
Climbing (s)
60
40
20
0
**
con
sham
PSND
PSD
e
Sucrose preference (%)
100
80
60
40
20
0
**
con
sham
PSND
PSD
f
Time in Center (s)
16
12
8
4
0
**
con
sham
PSND
PSD
g
Entries into Center (times)
60
40
20
0
**
con
sham
PSND
PSD

Figure S3

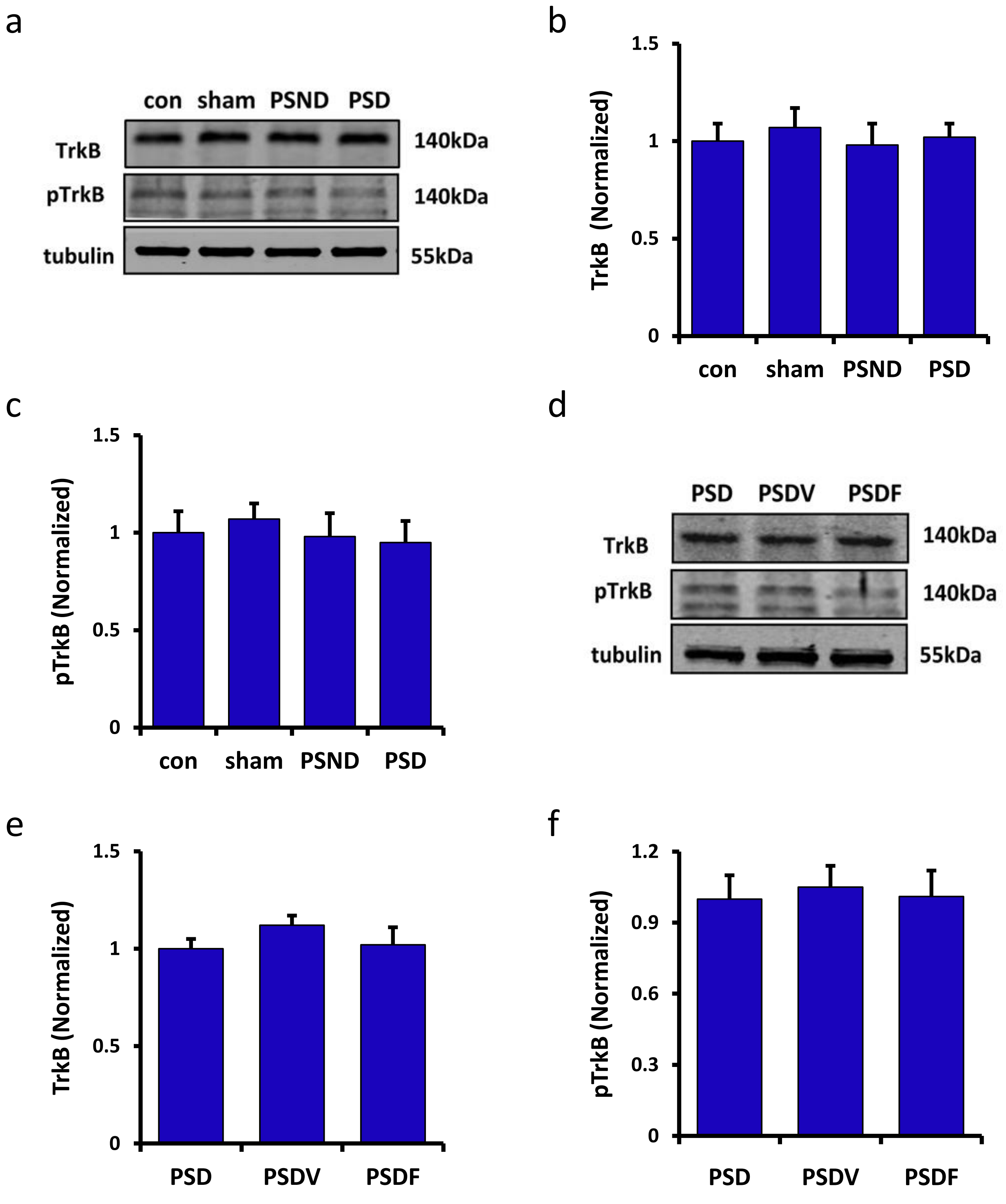
a
con
sham
PSND
PSD
TrkB
140kDa
pTrkB
140kDa
tubulin
55kDa
b
TrkB (Normalized)
1.5
1
0.5
0
con
sham
PSND
PSD
c
pTrkB (Normalized)
1.5
1
0.5
0
con
sham
PSND
PSD
d
PSD
PSDV
PSDF
TrkB
140kDa
pTrkB
140kDa
tubulin
55kDa
e
TrkB (Normalized)
1.5
1
0.5
0
PSD
PSDV
PSDF
f
pTrkB (Normalized)
1.2
0.9
0.6
0.3
0
PSD
PSDV
PSDF

Figure S4

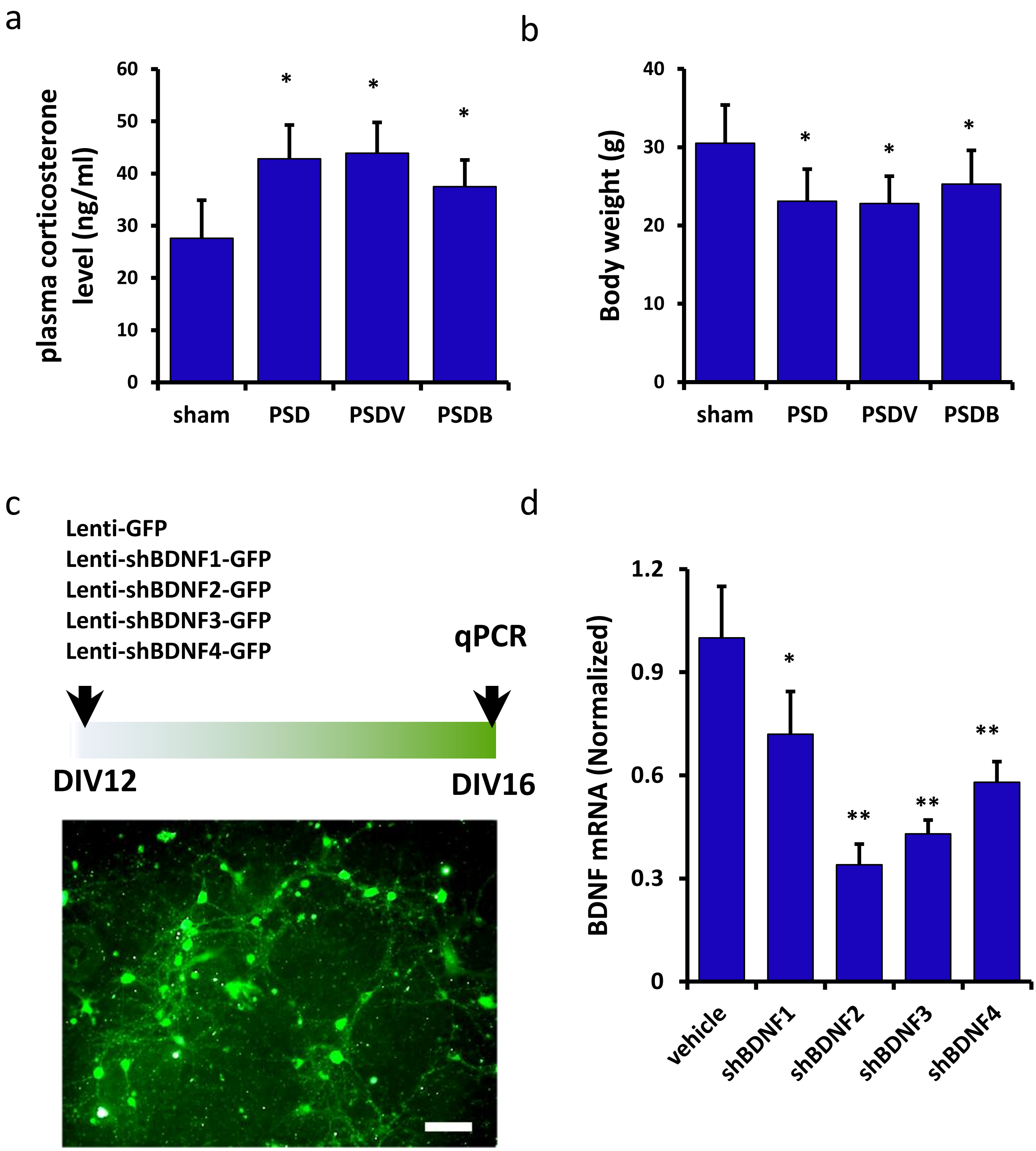
a
plasma corticosterone level (ng/ml)
0
10
20
30
40
50
60
*
*
*
sham
PSD
PSDV
PSDB
b
Body weight (g)
0
10
20
30
40
*
*
*
sham
PSD
PSDV
PSDB
c
Lenti-GFP
Lenti-shBDNF1-GFP
Lenti-shBDNF2-GFP
Lenti-shBDNF3-GFP
Lenti-shBDNF4-GFP
qPCR
DIV12
DIV16
d
BDNF mRNA (Normalized)
0
0.3
0.6
0.9
1.2
*
**
**
**
vehicle
shBDNF1
shBDNF2
shBDNF3
shBDNF4

Figure S5

Figure 2b

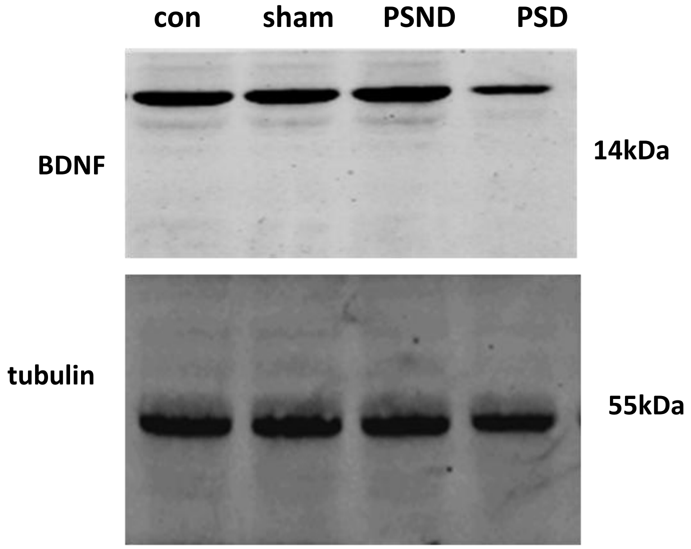

Figure 2d

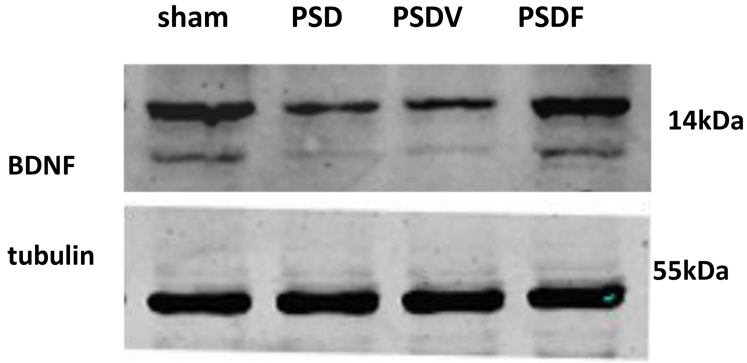

Figure 4c

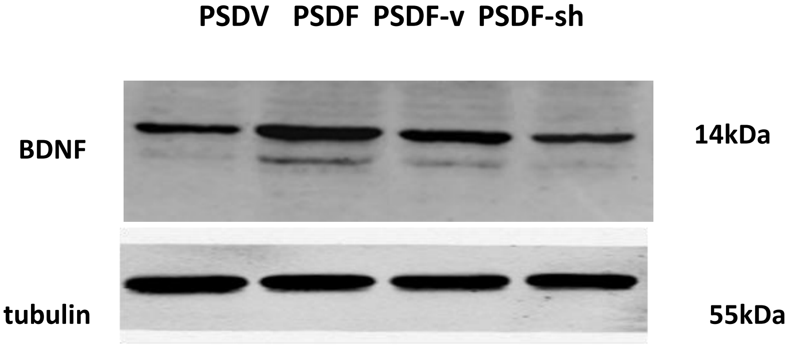

Figure 6b

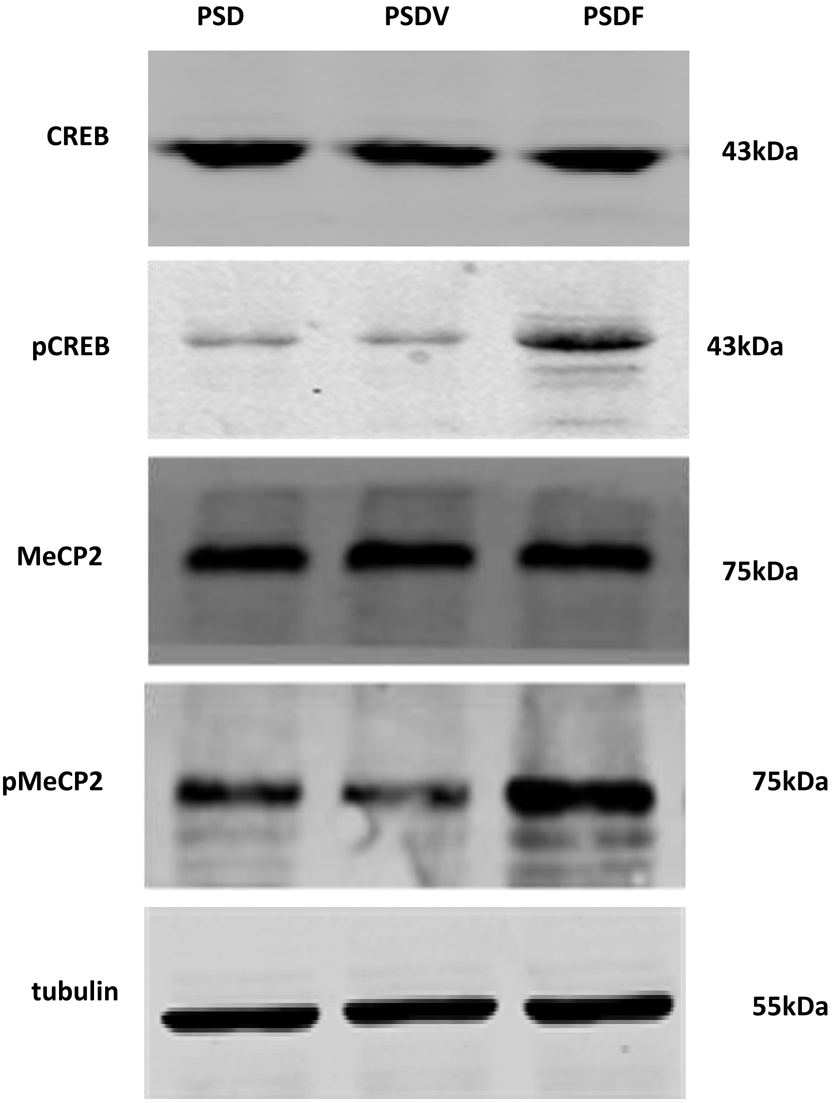

Figure 6g

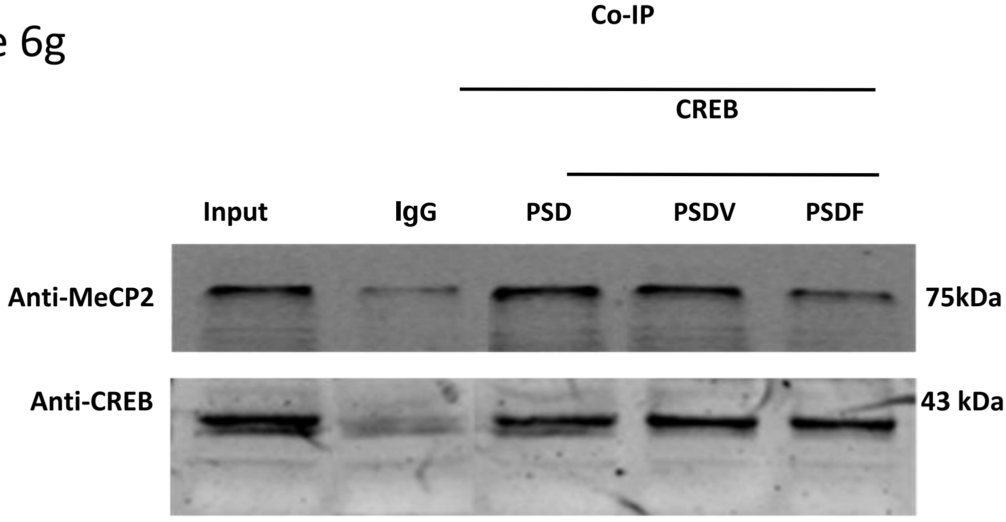

Figure 7a

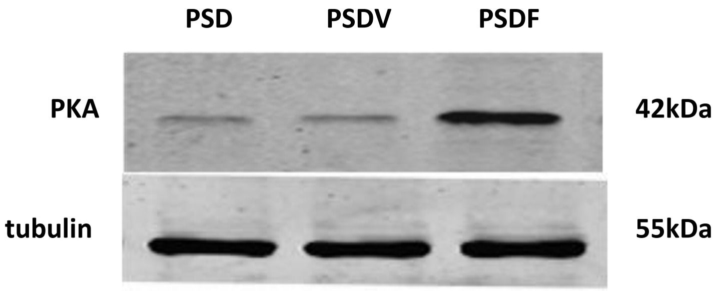

Figure 7b

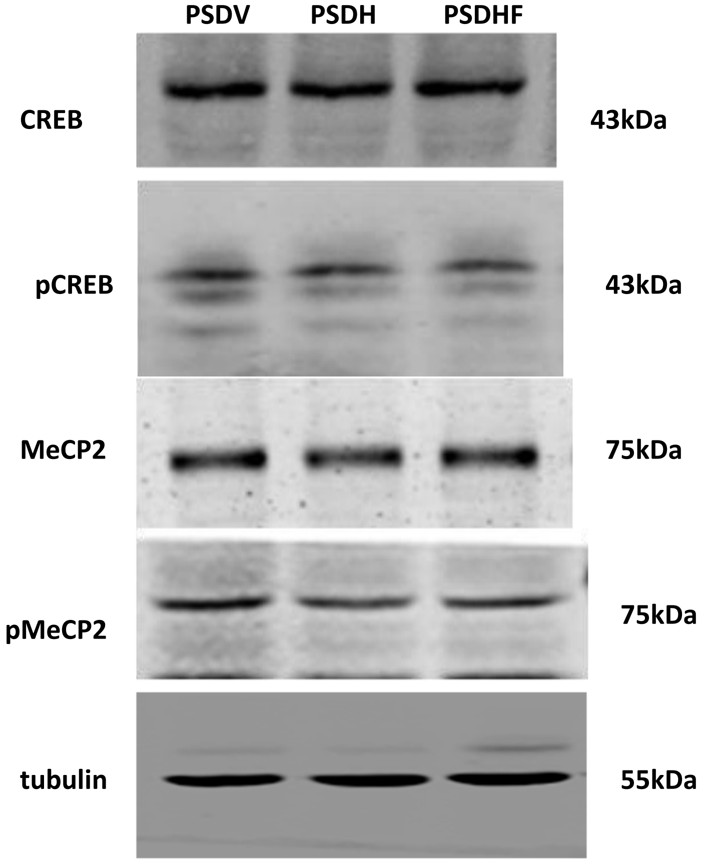

Figure 7g

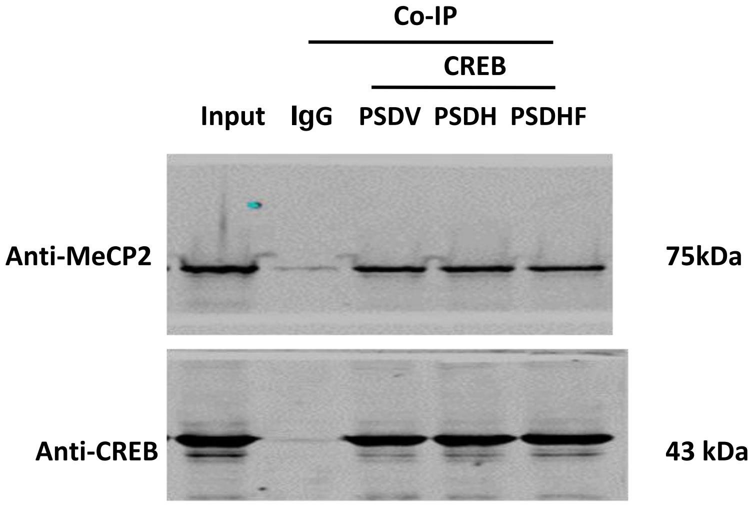

Figure S3a

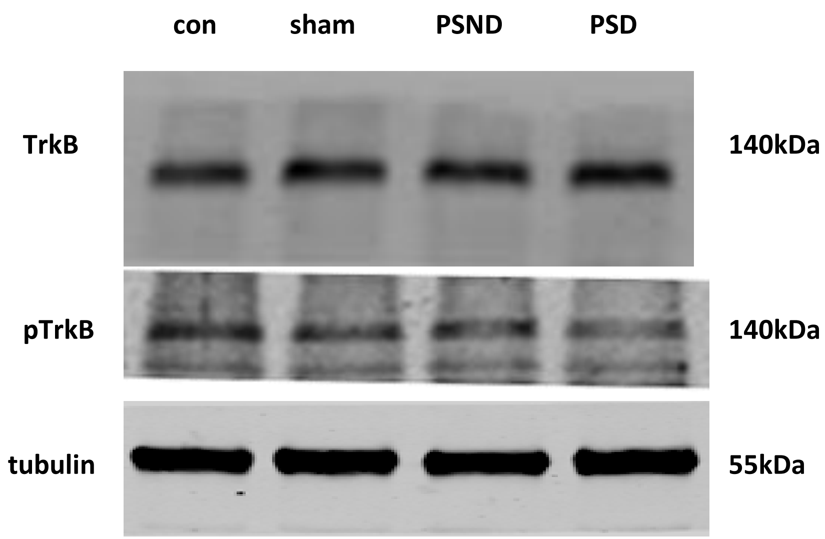

Figure S3d

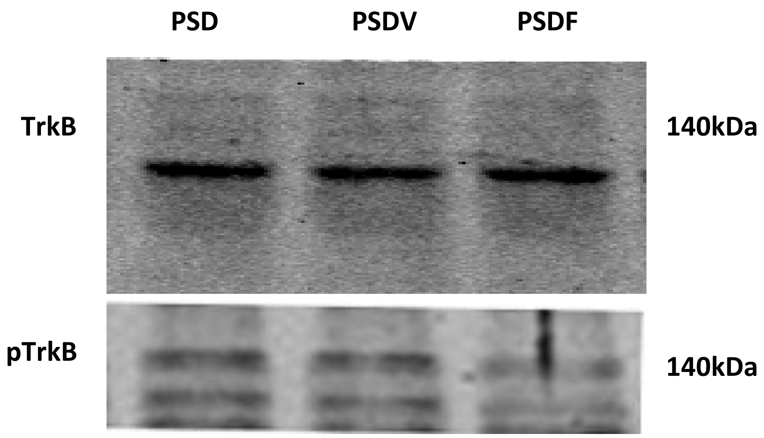

Supplement: Supplementary file 1 — supplementary information [file 41598_2017_13929_MOESM1_ESM.pdf]
